# MM_3_: Multimodal framework for regional-scale quantitative landslide risk analysis

**DOI:** 10.1016/j.mex.2025.103218

**Published:** 2025-02-18

**Authors:** William Pollock, Joseph Wartman

**Affiliations:** a Shannon & Wilson, Inc., 400N 34th St., Suite 100, Seattle, WA 98103, United States; b Department of Civil & Environmental Engineering, University of Washington, 201 More Hall, Seattle, WA 98195, United States

**Keywords:** Mass-wasting, Rockfall, Hazard, Risk assessment, Vulnerability, Coseismic, Precipitation-induced, Land use, Probabilistic analysis, Debris flow, *MM_3_: Multimodal framework for regional-scale quantitative landslide risk analysis*

## Abstract

Quantified estimates of landslide consequences in space and time (risk) facilitate rational land use decisions such as zoning for new development, protecting existing communities, allocating finite resources, designing mitigation works, and educating the public about natural hazards. Probabilistic landslide risk analysis (PLRA) should include all landslide modes, magnitudes, and triggering scenarios that could credibly cause harm and is most useful on a regional scale where landslide risk at a location can be compared across a broader area and in the context of other natural and anthropogenic sources of risk. However, to date, no readily transferable, regional-scale method for PLRA exists. In this work, we expand an existing deterministic multimodal method for landslide risk analysis developed in the country of Lebanon into a linked framework of code-based modules that are location-agnostic and computationally efficient for regional end-to-end risk estimation.

• Use of near-global, remote-sensing-based inputs enables risk estimates almost anywhere in the world
• Modular computational framework facilitates upgrades of component models as new research becomes available
• Probabilistic implementation through a Monte Carlo approach

## Graphical abstract

**Figure:**
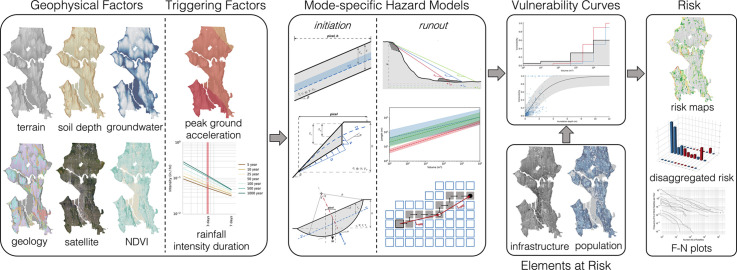

Specifications table

**Table:** 

Subject area:	Engineering
More specific subject area:	Landslide Risk
Name of your method:	*MM_3_: Multimodal framework for regional-scale quantitative landslide risk analysis*
Name and reference of original method:	Multimodal method for landslide risk analysis Pollock, W., Grant, A., Wartman, J., Abou-Jaoude, G. (2019). Multimodal method for landslide risk analysis. *MethodsX*, 6, 827–836. 10.1016/j.mex.2019.04.012.
Resource availability	*NA*

## Background

Rational land use decisions require knowledge of hazard and risk [1]. Deciding whether a location is “safe enough,” whether to allow development in one location but not another, how limited resources should be allocated, etc. are facilitated by quantitative risk analysis. Although methods for quantitative risk analysis of natural hazards such as earthquakes, flooding, windstorms, and hurricanes are established and currently used for land management, no comparable method exists for end-to-end risk analysis for landslides. This is due to the distinctive nature of landslides. Landslides are exceptionally varied in space, time, and behavior. Unlike other natural hazards, landslides are simultaneously widespread, found across virtually any sloping terrain, and hyper-localized, with individual events occurring on the scale of a single slope. Landslides may occur as a secondary hazard of a distinct trigger (e.g., an earthquake, rainstorm, flood, etc.) or as a product of gradual processes such as soil creep, slope toe erosion, or vegetative root decay. Furthermore, landslides—and their associated consequences—occur in numerous modes, ranging from channelized, fluid debris flows and massive, rapid debris avalanches, to individual falling rock blocks and slow-moving, episodic slumps.

Although there is general consensus in the literature about the conceptual framework to perform landslide risk analysis (e.g., [[2], [3], [4]]), the scale and diversity of landslide hazards limits the scope and transferability of existing methods. Numerous models have been proposed to represent individual components of landslide risk. However, these models rarely integrate through coordinated inputs and outputs (I/O). To our knowledge, no comprehensive framework exists that combines existing component models to calculate total risk in a repeatable and transferable manner.

## Method details

### Quantitative landslide risk analysis

**Fig. 1:**
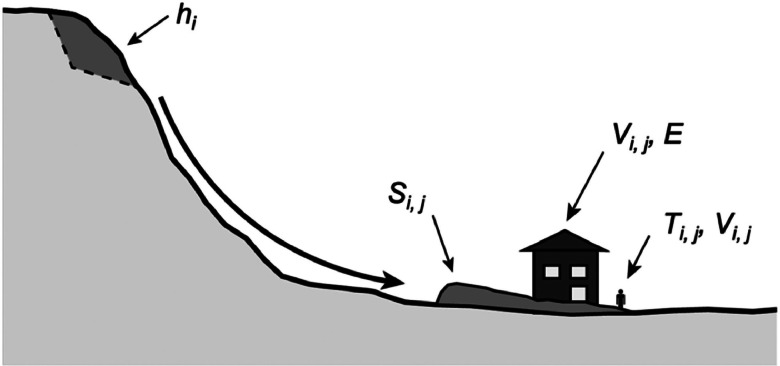
Elements of the risk equation. Modified after van Westen et al. [6].

Landslide risk analysis involves estimating the consequences of landslides in time and space to elements at risk within an area of interest. Risk can be quantified through the summation of a series of statistical probabilities describing independent landslide events and the element(s) of interest which they impact:

(1) $$R_{j\;\left(Loss\;of\;Life\right)}=\sum_{1}^{n}\;h_{i}\;x\;S_{i,j}\;x\;T_{i,j}\;x\;V_{i,j}$$

and

(2) $$R_{j\;\left(Infrastructure\right)}=\sum_{1}^{n}\;h_{i}\;x\;S_{i,j}\;x\;T_{i,j}\;x\;V_{i,j}\;x\;E$$

where *R_j_* is the annual probability of death of individual *j* (also known in the literature as PDI) or the annual monetary loss due to landslide damage to building *j; h_i_* is the annual incremental probability of landslide scenario *i* occurring out of *n* total scenarios (*initiation hazard*); *S_i,j_* is the spatial probability of landslide *i* reaching a location commonly occupied by the element at risk, *j* (*runout*); *T_i,j_* is the spatial-temporal probability of the element at risk being in the location affected by the landslide at the time of occurrence (*exposure*); *V_i,j_* is the probability of death or degree of damage if the element at risk is present and impacted by the landslide (*vulnerability*); and *E* is the total monetary value for infrastructural elements at risk [5,6]. Fig. 1 illustrates the elements of the risk equation. As in Eq. (2), a multiplier, *E*, representing the resident population, can be added to Eq. (1) if the loss-of-life risk is desired for a single unit location with multiple occupants (e.g., building, vehicle, road segment, neighborhood, etc.) where the runout, exposure, and vulnerability components of risk are reasonably the same for each individual. In the case of static infrastructure, the exposure, *T_i,j_*, equals one.

Risk is calculated uniquely for each credible landslide scenario 1 to *n*, differentiated by landslide type and magnitude, for a given element at risk. Then the products are summed to produce total risk. Flow behavior such as channel avulsion or flow bifurcation, which could be missed using traditional runout models, can be incorporated by adding sub-scenarios with conditional probabilities that sum to one [7]. In the case of an indoor population, the vulnerability, *V_p_*, in Eq. (1) may include the vulnerability of the occupied building, *V_i_*, as well as conditional, behavioral factors such as the ability to take evasive action. While less common, risk to environmental features and economic activity may also be included in risk analyses. Quantifying losses to these elements is more challenging, as it may involve making subjective estimates of value and considering the time after the event until the system returns to its pre-event capacity.

### Multimodal framework for regional-scale quantitative landslide risk analysis

Landslide hazard is quantified using statistical or physically-based approaches [2]. While physically-based models are data-intensive on regional scales and significantly simplify landslide geometry and processes, they provide key advantages over statistical models in quantitative risk assessment. Unlike statistical models, physically-based models are not dependent on the stasis of present environmental conditions [6]. Furthermore, because they capture the underlying physical processes that lead to landslide failures, physically-based models are not necessarily limited to the area in which they were developed but are applicable across geologic, physiographic, and climatic regions. Since a key goal of risk assessment is to disaggregate the risk contribution of different triggering scenarios, such as storm precipitation or earthquake shaking, statistical models are less useful for risk assessment when triggering data is not linked to the landslide record. Finally, unlike statistical models, physically-based models do not require detailed landslide inventories, which are costly to develop, often incomplete, and too short to capture large, infrequent events [8,9].

In this work, we introduce the third generation of the multimodal method for landslide hazard and risk analysis, herein referred to as “MM_3_,” which updates and expands the first (“MM_1_”) and second (“MM_2_”) generation models introduced by Grant et al. [10] and Pollock et al. [11], respectively. The most significant updates in MM_3_ include (1) the incorporation of a physically-based model for groundwater rise due to rainfall; (2) improvements to the mode-specific landslide initiation models; (3) dynamic runout modeling, including rockfall fragmentation; and (4) intensity-dependent empirical vulnerability functions. We explicitly account for uncertainty in the input parameters and propagate it through to final risk using a Monte Carlo approach. Additionally, the governing equations of the updated multimodal method have been implemented into a modular, Python-based computational framework. In this framework, each module communicates with others through memory objects that can optionally be written to geospatial files, such as GeoTIFFs or GeoPackages. This flexible computational approach allows individual modules (e.g., water table depth, debris flow runout, infrastructural vulnerability to rockfall, etc.) to be exchanged and their outputs checked at any stage of the risk calculation. The application of this computational platform is demonstrated in Seattle, Washington, USA, by Pollock [12]. Pollock [12] provided a sensitivity analysis of MM_3_ to variations in user-selected model inputs and compared landslide hazard estimated with MM_3_ to observed landslide event data.

**Fig. 2:**
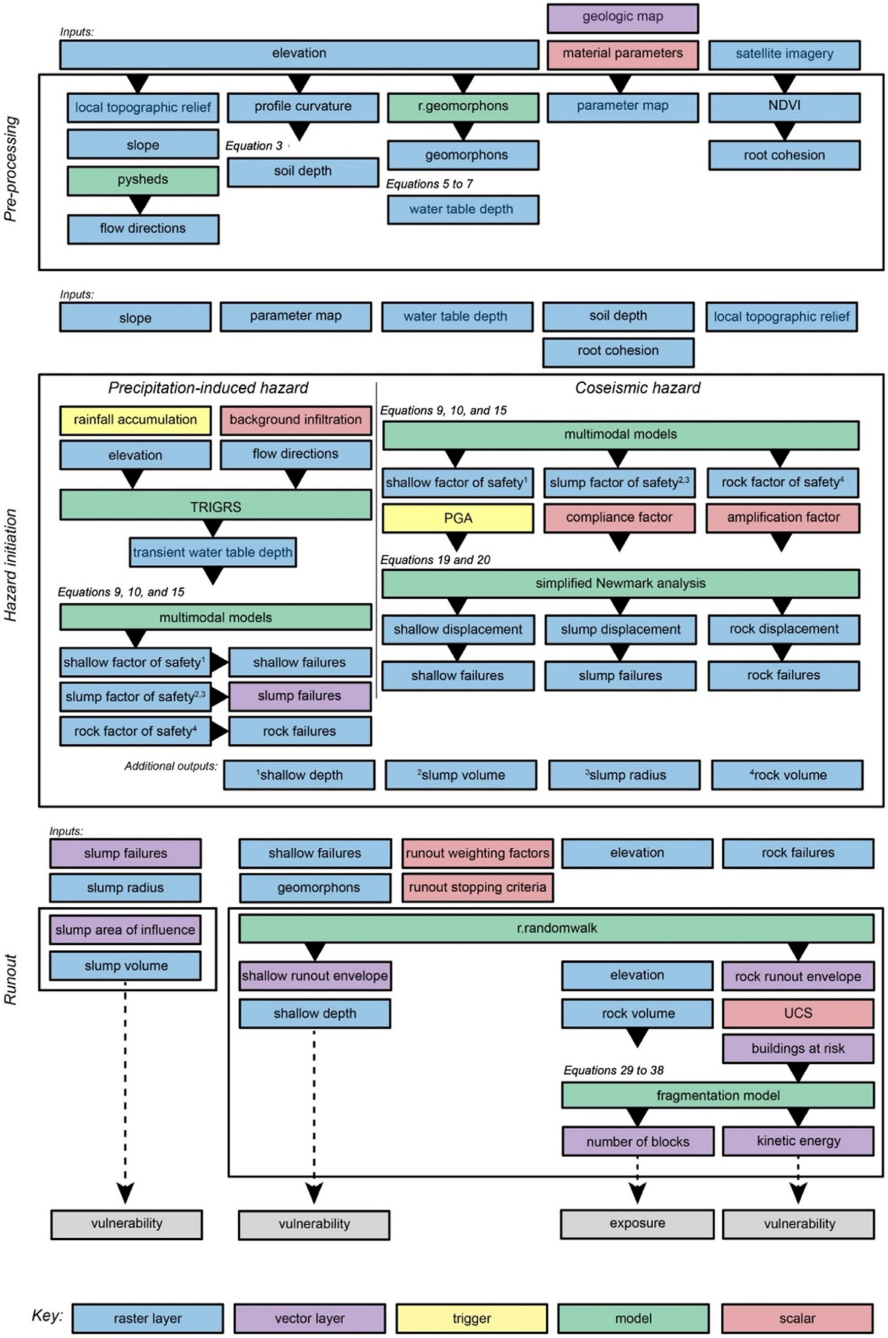
Workflow and component modules of MM_3_, including the pre-processing, hazard initiation, and runout modelling steps.

The following sections describe the modules and workflow of MM_3_, which are visualized in Fig. 2. Applying the risk equation (Eq. (1)) to real-world scenarios is complex [6]. A significant body of research is associated with each component of risk individually, as well as risk analysis as a whole. In this work, we do not attempt to a full synthesis of available component models, but instead focus on the component models chosen or developed into modules in MM_3_. For a comprehensive overview of the entire risk management process, we refer interested readers to detailed review papers [[2], [13], [14], [15], [16]] and the deliverables of the Safeland Project (https://www.ngi.no/eng/Projects/SafeLand). Additionally, readers can find reviews of individual risk components, such as runout [17] and vulnerability [18,19].

We utilize published component models where available and appropriate to the scale and purpose of MM_3_. We selected existing component models for inclusion in MM_3_ based on multiple criteria: (1) open-source and transparently documented, (2) computationally efficient at regional scales, and (3) physically based or trained on large global datasets that can be reapplied broadly (with care) for screening-level assessments. Criterion (2) was of particular importance because many state-of-the-art models are, at present, too computationally intensive to be implemented probabilistically or across large domains. Additionally, they require detailed, site-specific parameterization or calibration that rarely exists outside of a single case study. We created modules consisting of existing software wrapped in Python “helper” functions to automate the I/O processes that would usually be performed manually in a graphical user interface and to unify the model outputs for use in subsequent steps of the risk calculation. Where existing software meeting the above criteria did not exist, we coded models presented in the literature or developed our own, as described in the following sections.

#### Landslide modes

**Fig. 3:**
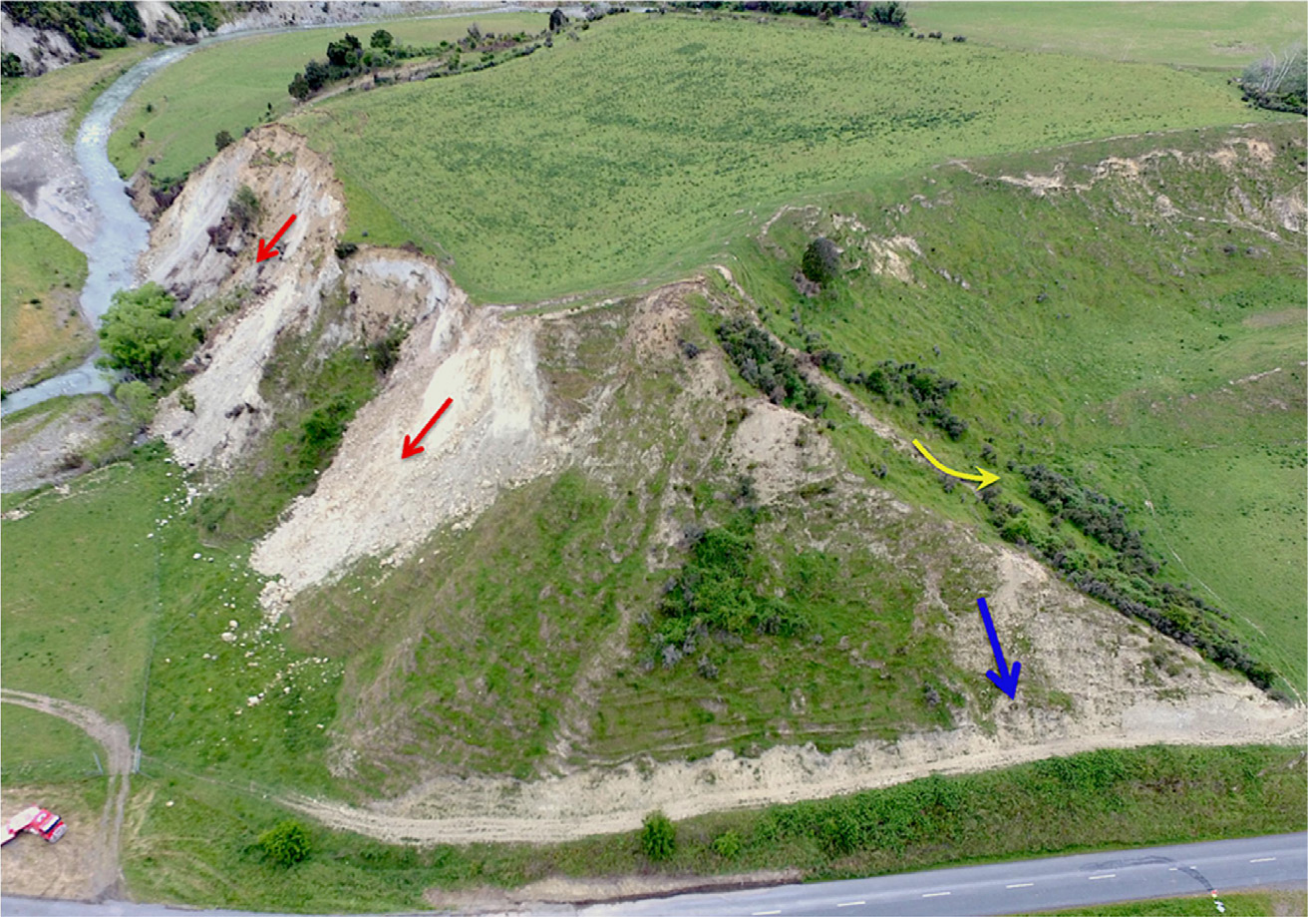
Coseismic rock avalanches (*red*), a shallow disrupted slide (*blue*) and a deep-seated rotational slump (*yellow*) triggered by the 2016 Kaikoura earthquake in New Zealand.

Traditional regional-scale landslide hazard and risk analyses typically reduce the diverse variety of landslide types and processes (“modes”) into a simplistic infinite slope approach. However, unique modes of landsliding cause unique consequences to the human environment [5,6]. For instance, slow, deep-seated landslides can be catastrophic for infrastructure while posing little danger to humans, while rapid shallow flows pose greater risk to humans. Hungr et al. [20] revised the traditional Varnes’ classification to describe 32 distinctive modes of landsliding. After Grant et al. [10], we simplify this classification to three basic landslide modes corresponding to the initial failure process: 1) rockfalls and avalanches; 2) shallow, planar soil slides; and 3) deep-seated, rotational slumps in soil and rock. All three modes may be caused by seismic- or precipitation-triggering, and in some cases, they may be collocated, as shown in Fig. 3.

**Fig. 4:**
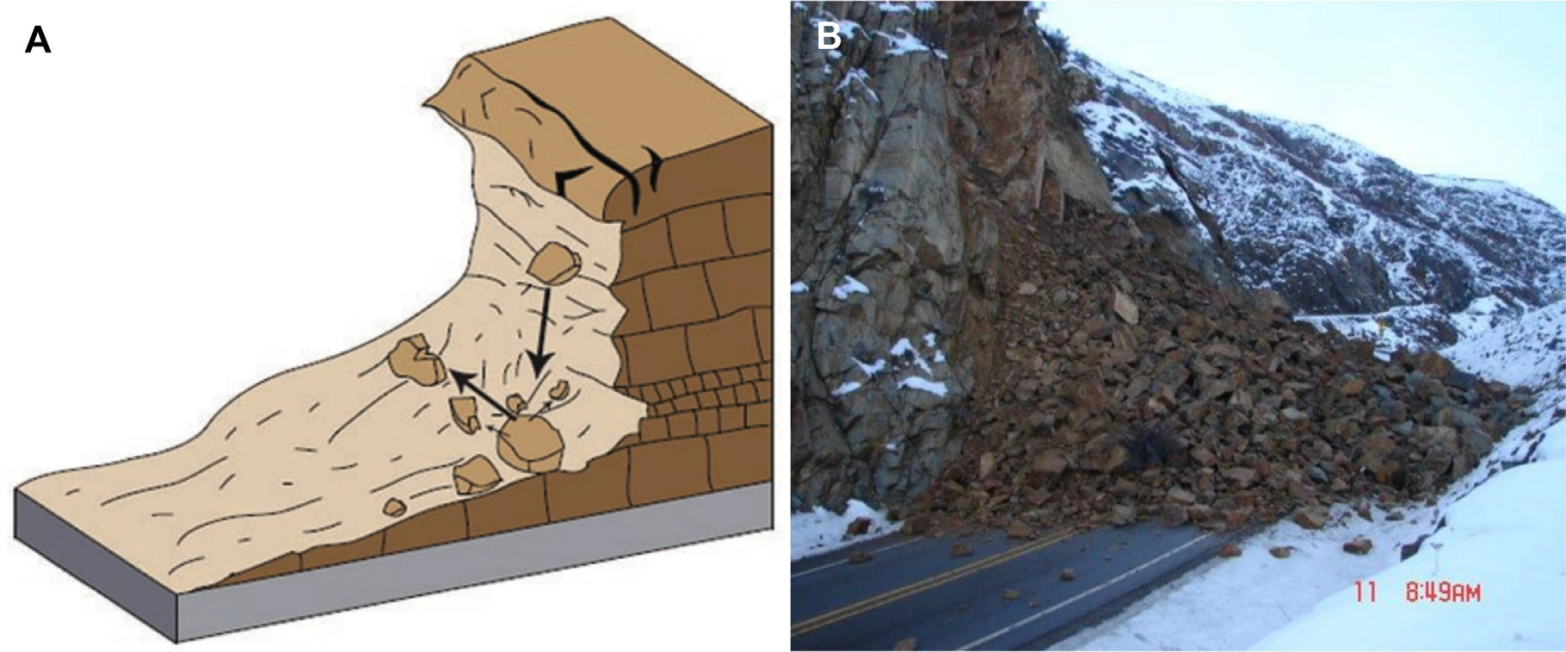
Rockfall and rock avalanche. (*A*) Simplified diagram and (*B*) rock avalanche near [22]. Figure courtesy of the U.S. Geological Survey [23], based on Cruden and Varnes [21]. Image: Washington State Department of Transportation (WSDOT), “US 2 – Pine Canyon East” (License: CC BY-NC—ND 2.0).

**Fig. 5:**
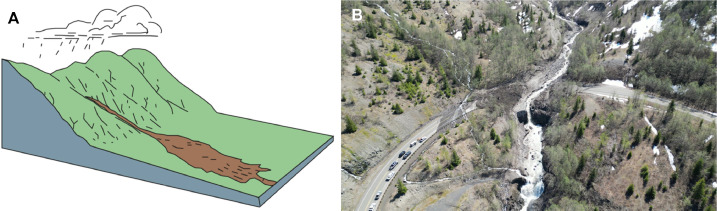
Soil slide transitioning into a debris flow. (*A*) Simplified diagram and (*B*) debris flows near Johnston Ridge, Washington, 2008. Figure courtesy of the U.S. Geological Survey [23], based on Cruden and Varnes [21]. Image: WSDOT, “SR 504 – Spirit Lake Outlet Bridge – Washout” (License: CC BY-NC—ND 2.0).

**Fig. 6:**
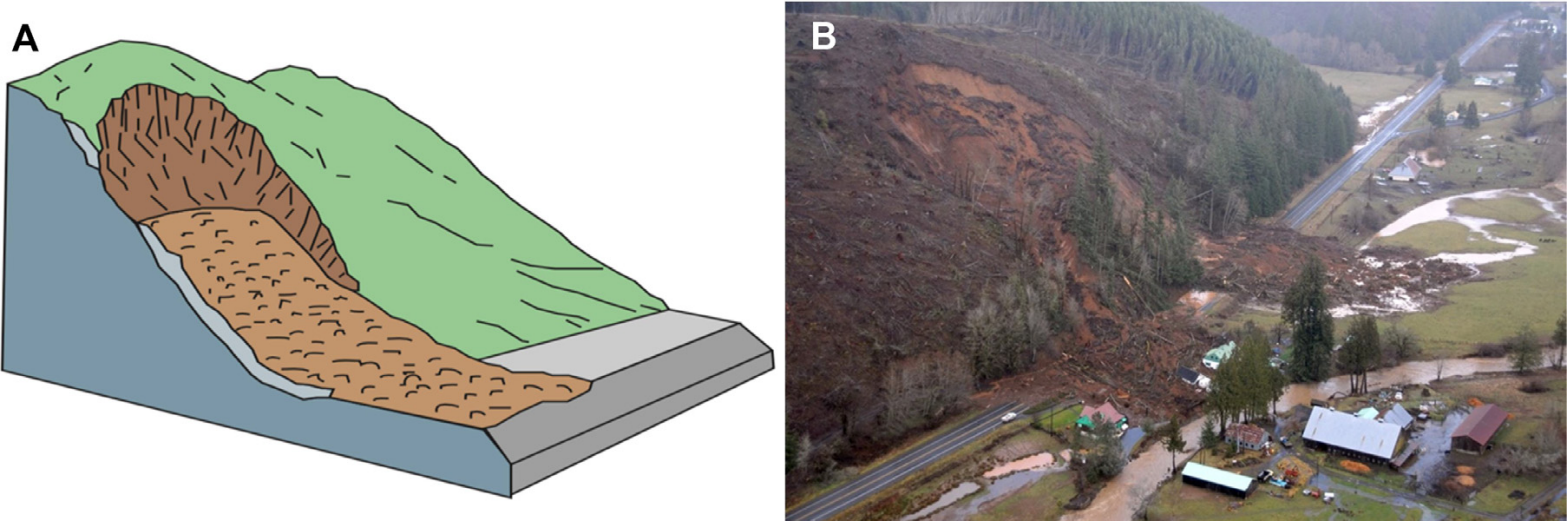
Soil slide transitioning into a debris avalanche. (*A*) Simplified diagram and (*B*) debris avalanche in Pe Ell, Washington, 2007. Figure courtesy of the U.S. Geological Survey [23], based on Cruden and Varnes [21]. Image: WSDOT, “SR 6 mudslide – near Pe Ell” (License: CC BY-NC—ND 2.0).

We further differentiate between landslide modes on the basis of post-failure runout behavior. Rockfalls and rock slides can disintegrate into rock avalanches, and in the presence of abundant water, shallow soil slides can mobilize into debris flows (channelized) or debris avalanches (un-channelized). Table 1 summarizes the landslide typology used in MM_3_. Figs. 4 **to** 7 provide examples of the landslide modes corresponding to the definitions in Table 2, Figs. 5, 6.

**Table 1:** Landslide typology used in MM_3_. Velocity classes after Cruden and Varnes [21].

*Trigger*	*Mode number, material, and initial failure type*	*Sub-mode based on runout behavior*	*Velocity class*
seismic	(1) rockfall	(1a) rock avalanche	very rapid to extremely rapid
	(2) shallow disrupted soil slide		very rapid to extremely rapid
	(3) soil/rock rotational slump		slow to very rapid
precipitation	(1) rockfall	(1a) rock avalanche	very rapid to extremely rapid
	(2) shallow soil slide	(2a) confined debris flow	very rapid to extremely rapid
		(2b) unconfined debris avalanche	very rapid to extremely rapid
	(3) soil/rock rotational slump		very slow to very rapid

**Table 2:** Definitions for the landslide modes used in MM_3_ and introduced in Table 1. Definitions and terminology adopted from Hungr et al. [20] and Keefer [24].

*Mode*	*description*
(1) rockfall	Small failures occur as the detachment, fall, rolling, and bouncing of rock fragments. May occur singly or in clusters, but with little dynamic interaction between the most mobile moving fragments, which interact mainly with the substrate. Fragments can break during impacts. Larger, structurally-controlled rock slides occur as a sliding mass of rock on a planar rupture surface.
(1a) rock avalanche	Extremely rapid, massive, flow-like motion of fragmented rock from a large rock slide or rockfall
(2) disrupted soil slide	Sliding of a mass of granular material on a shallow, planar surface parallel with the ground. Usually, the sliding mass is a veneer of colluvium, weathered soil, or pyroclastic deposits sliding over a stronger substrate.
(2a) debris flow	Very rapid to extremely rapid surging flow of saturated debris in a steep channel. Strong entrainment of material and water from the flow path.
(2b) debris avalanche	Very rapid to extremely rapid shallow flow of partially or fully saturated debris on a steep slope, without confinement in an established channel. Sometimes called a “flowslide.”
(3) rotational slump	Sliding of a mass of homogeneous and usually cohesive soil or weak, non-structurally controlled rock on a semi-spherical or ellipsoidal rupture surface. Prominent main scarp and back-tilted landslide head. Normally slow to rapid.

**Fig. 7:**
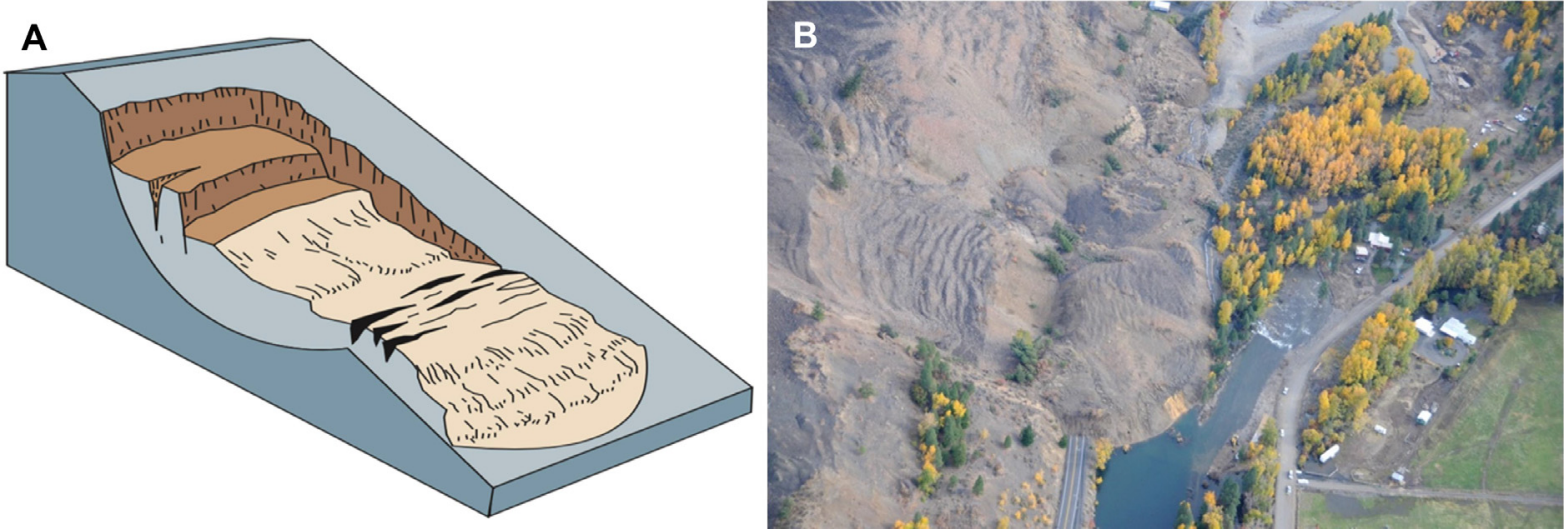
Rotational slump. (*A*) Simplified diagram and (*B*) deep-seated, rotational slump near Naches, Washington, 2009. Figure courtesy of the U.S. Geological Survey [23], based on Cruden and Varnes [21]. Image: WSDOT, “SR 410 Nile Valley Landslide – Oct. 2009″ (License: CC BY-NC—ND 2.0).

#### Terrain-based susceptibility

Statistical studies have found a correlation between topographic slope and landslide mode (e.g., [25,26]). To limit excess computation, we divide the terrain into slope-based zones susceptible to each failure mode in Table 1 in which mode-specific physical models are applied (Table 3). We defined slope ranges of 15° to 50°, 10° to 35°, and greater than 35° as susceptible to shallow planar slides, rotational slumps, and rockfalls, respectively [10,27]. We consider slopes shallower than 10° unlikely to fail in any landslide mode [28].

**Table 3:** Slope ranges for mode-specific susceptible zones.

*Mode*	*Slope range (degrees)*
(1) rockfall	35 to 90
(2) shallow soil slides	15 to 50
(3) soil / rock slumps	10 to 35

#### Input data

##### Elevation

The primary input for MM_3_ is a digital elevation model (DEM). The DEM forms the basis of five secondary inputs. (1) Local relief is measured within a moving circular window of user-defined size based on the scale of major topographic features within the area of interest. Topographic wavelength or stream network analyses can be used to semi-automate the selection of an appropriate window size, although such methods may obscure critical landslide-forming terrain features if applied blindly (e.g., glacial benches or inner gorges), and for this reason we have not included automated selection of window size in MM_3_. However, MM_3_ is generally insensitive to local relief window size [12]. (2) Slope is calculated both at individual cells and as an average within a moving window one quarter the size of the window for local relief. This average slope, representing a “smoothed” topographic surface, is used in the rock and rotational slump models to prevent overpredicting instability from hyper-local oversteepening. (3) Topographic profile curvature is used to estimate soil depth, as described in Section 2.3.2. (4) As a preprocessing step for the precipitation-induced failure modules, the DEM is pit-filled and used to extract flow directions with the Python-based model pysheds [29]. Finally, (5) the DEM is used to perform a basic morphometric analysis with the GRASS Geographical Information System (GIS) model r.geomorphons [30] which delineates the spatial distribution of confined or “channelized” topography for runout analyses.

##### Soil depth

Depth of mobile regolith (“soil depth”) is an important input parameter to physically-based landslide models, as it typically defines the depth of shallow failure over a stronger substrate. Soil depth is frequently assumed as a uniform mantle across a landscape owing to the scarcity of soil depth surveys [31]. However, soil depth varies as a function of inter-related factors such as topography, parent material, climate, and vegetation, and the assumption of uniformity may lead to significant under- or over-prediction of landsliding [32,33]. Statistical approaches have been developed relating soil depth to easily-measured topographic variables such as slope, curvature, wetness index, contributing area, and distance from hilltop ( [33]; Catani et al., 2010). Patton et al. [34] demonstrate that a profile curvature-based prediction relationship performed reasonably well across multiple catchments with a variety of underlying lithologies, climatic regimes, and topographic characteristics:

(3) $$d=\;\left(\frac{\Delta d}{\Delta C}\right)+d_{C=0}$$

where *d* is the soil depth, d_C=0_ is the average soil depth measured at locations of zero curvature, and *Δd/ΔC* can be calculated from the regression:

(4) $$\frac{\Delta d}{\Delta C}=\;-446.3*\;\sigma_{C}+30.3$$

where *σ_C_* is the standard deviation of curvature [m^-1^]. Use of Eq. (3) requires only the soil depth on flat terrain, which can be estimated from a limited number of field explorations within the area of interest.

##### Water table depth

The water table is typically a repressed imitation of the land surface [35]. Peck and Payne [36] propose a general regression between the land surface elevation (LSE) and water table elevation (WTE) for determining the elevation of the water table over regional scales:

(5) WTE=0.9505*LSE−1.9955

where both WTE and LSE are in meters. Where site-specific water table elevation is available, a site-specific regression can be produced following the method of Peck and Payne [36], with the added constraint of the water table being coincident with the land surface at sea level (if present in the study area):

(6) WTE=a*LSE

Water table depth (WTD) is the difference between the land and water surface elevations:

(7) WTD=LSE−WTE

##### Satellite imagery

The presence and type of vegetation influences the location of shallow precipitation-induced landslides, primarily due to its contribution to effective soil cohesion through a root network [37]. The presence and relative health of vegetation can be rapidly assessed through multispectral satellite imagery. Grant et al. [10] found a strong relationship between shallow coseismic landslides and the relative normalized difference vegetation index (NDVI). NDVI is calculated from the red and near infrared (NIR) bands:

(8) $$NDVI=\;\frac{NIR-\;red}{NIR+red}$$

where low values correspond to bare soil and values approaching one correspond to dense, healthy vegetation [38,39]. By examining the distribution of NDVI values in areas of observable vegetation classes (e.g., mature forest, grasslands, etc.) and combining it with knowledge of the local vegetation, discrete classes of root cohesion can be assigned across the landscape. Schmidt et al. [40] and Cislaghi et al. [41] provide root cohesion values associated with common vegetation types. In MM_3_ we adopt three classes of root cohesion—none, moderate, and high—although any number of classes could be used. Examples are given in Pollock [12].

##### Geologic map and soil strength parameters

Soil and rock units are identified on the basis of geologic mapping. Material strength and hydrologic parameters are assigned to each geologic unit. Vertical heterogeneity is not explicitly taken into account in MM_3_, although unique input parameters are assigned to mobile regolith and the underlying substrate. We recommend consolidating geologic maps into a smaller number of similar “engineering geologic units” (EGUs) depending on the detail of published parameter values relevant to the study area (e.g., [42]). Required parameters include cohesion, friction angle, unit weight, hydraulic diffusivity, vertical conductivity, and parameters of soil-water characteristic curves [43]. Development of realistic ranges for material parameters constitutes the most data-intensive parameterization step in MM_3_.

The assumption of vertical homogeneity belies the stratigraphic control of the location, shape, volume, and runout behavior evident in many deep-seated landslides, especially in glacially altered terrain (e.g., [44]). However, three-dimensional geologic models require intensive exploration programs and are rarely developed even at site-specific scales. In some heavily developed urban areas, such as the city of Seattle, Washington, multi-decadal subsurface exploration databases compiled from public and private sectors permit three-dimensional subsurface modeling at a local scale (e.g., [45]). However, this is the exception, not the norm. Although multiple, pixel-based deep-seated slope stability software packages that are capable of incorporating three-dimensional subsurface geometry are current available (e.g., [46,47]), we have elected to use the simplified stability modules described in Sections 2.4 and 2.5 to maintain maximum transferability of MM_3_ until robust regional subsurface datasets are developed. This decision is consistent with use of MM_3_ as a screening tool intended to guide and complement, rather than replace, site-specific studies.

Surficial geology maps necessarily simplify complex, transitional, and ambiguous geologic realities into discrete two-dimensional boundaries. Uncertainty in geologic maps comes from at least three sources [48]. *Conceptual* uncertainty describes the artificiality of representing a spatially continuous or irregular transition between different units by a line. Such divisions will often vary between surveyors. *Interpretation* uncertainty exists because evidence of a geologic contact is not observable everywhere due to surficial cover, land-access permissions, or the practical resource limitations of a field campaign. Thus, the location of geologic boundaries is also subject to the interpretation of the surveyor. The final type of uncertainty is *scale-dependent* uncertainty. A map line drawn at 1:24,000 scale with a 0.5 mm pen will represent a 12 m band on the ground, within which the location of the geologic boundary it represents is uncertain.

These three sources of uncertainty are especially relevant for landslide risk analysis when key parameters, such as soil strength, are assigned on the basis of mapped geologic units. Twelve (or more) meters of uncertainty may not matter in undeveloped land but will be very important to a homeowner whose property is on the boundary of a particularly weak geologic unit.

**Fig. 8:**
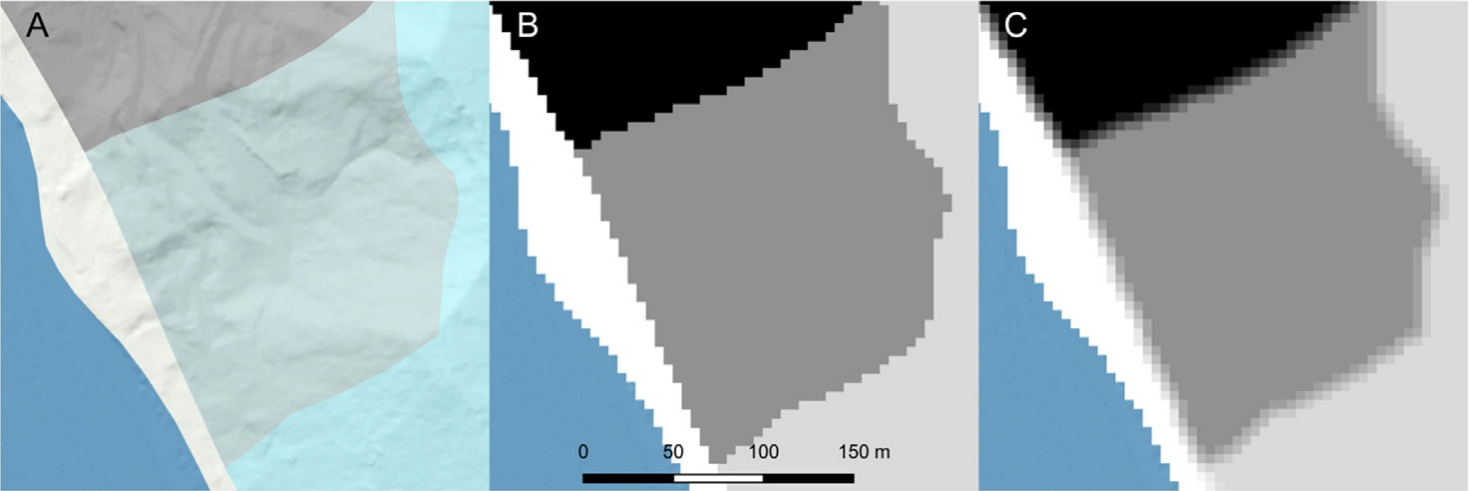
Starting with a vector-based geologic map (*A*), material parameters are assigned to mapped units at the pixel size of the underlying elevation model (*B*). The borders between units are then smoothed using a circular moving window based on the horizontal map accuracy and resolution (*C*).

In MM_3_, we only address scale-dependent uncertainty. Conceptual and interpretation uncertainty will vary by map, location, and surveyor and are beyond the scope of this work, although some conceptual uncertainty is implicitly accounted for in our approach. The United States Geological Survey (USGS) standard for its map products is that 90 % of all points must fall within 1/50th of a map inch [49]. At a 1:24,000 scale this translates to 12.2 m. In MM_3_, the accuracy and scale of the adopted geologic map is a required input. This produces an accuracy window size (12.2 m in the example above) which is converted to the closest higher odd number of cells based on the DEM resolution. Values of friction angle and cohesion throughout the domain are then averaged within a circular moving window based on the number of cells to “smear” the parameters across geologic boundaries, preventing adjacent cells from having an artificially discrete transition between weak and strong materials (Fig. 8).

##### Distributions of material parameters

Where empirical distributions of parameters do not exist in a given area, distributions are assigned based on data from other regions. For instance, the mean and standard deviation of friction angle and cohesion may not be known. In such cases, mean values should be adopted from best-estimate values in published studies of similar geologic units and then increased as necessary to meet the limiting criteria of stability under static conditions. MM_3_ estimates standard deviations based on a coefficient of variation (COV) of 0.1 and 0.3 for friction angle and cohesion, respectively [[50], [51], [52], [53], [54]]. This method falls between a purely data-driven (empirical testing) approach and a logic tree approach in which the weights of medium, upper, and lower-bound strengths are fixed heuristically [54]. Friction angle is assumed to be normally distributed while cohesion and saturated hydraulic conductivity are assumed to be log-normally distributed [[51], [55]].

##### Triggering factors

Slope failures are caused by a combination of long-term conditioning processes (e.g., sediment production, stream erosion, weathering, etc.) and a short-term destabilizing “trigger” such as earthquake loading, pore pressure increase due to a rainstorm, or construction activity [5]*.* MM_3_ incorporates seismic and precipitation landslide triggers. Earthquake and rainfall scenarios must be reduced to quantitative maps of the factors directly related to landslide initiation (e.g., peak ground acceleration (PGA) or rainfall accumulation). These factor maps may be produced from measured earthquake or rainfall events in the historical record or developed through probabilistic hazard analyses. In the latter case, PGA or time-dependent rainfall intensities are statistically associated with a temporal frequency, most often in the form of a return period. A full risk curve considers every plausible pairing of magnitude and frequency of the triggering factor, as described by a magnitude-frequency (M-F) curve. However, in practice it is only feasible to consider representative, discrete events which approximate the full M-F curve [7]. What events are “representative” is defined by the user or can be selected to match local guidelines. For instance, on Washington State highways, the performance of most bridges is evaluated for the 210- and 1033-year return periods seismic events and 100- and 500-year flood events [56,57]. The risk analyst must decide the appropriate number of events to balance computational expense with a complete analysis appropriate to the study area in question and the data available.

##### Elements at risk

**Fig. 9:**
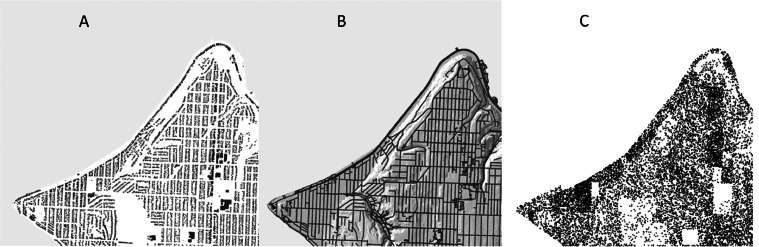
Examples of elements at risk in West Seattle, Washington State. (*A*) Building inventory [58], (*B*) road inventory [63], and (*C*) population by census block represented as points [64].

MM_3_ analyzes only the risk to physical elements, including human populations, commercial or residential structures, critical facilities, transportation networks, communication lines, and utilities (Fig. 9). While risk to economic activities may be derived from direct landslide damages to some or all of the elements above it is not directly considered in this framework. Environmental damage and losses of cultural or historical significance, while significant, are even more challenging to quantify objectively and are thus excluded from MM_3_. Human and infrastructural data can be represented either as a continuous raster or as discrete vector elements (e.g., [12,11]). Common sources of publicly available element-at-risk GIS data include city building databases (e.g., [[58], [59]]), crowed-sourced infrastructure repositories (e.g., OpenStreetMap: www.openstreetmap.org), national census data (e.g., U.S. Census Bureau: www.census.gov), satellite-derived infrastructure maps (e.g., [60]), global population density estimates (e.g., [61]), and global mapping of human built-up presence (e.g., [62]). These data sources should provide sufficient information, either as separate raster layers or vector feature attributes, for determining exposed population and value (see Eqs. (1) and (2)), such as number of individuals, number of building stories, material type, cost per area for repairs, appraised building value, etc.

#### Precipitation-induced landslide models

##### Transient water table depth

**Fig. 10:**
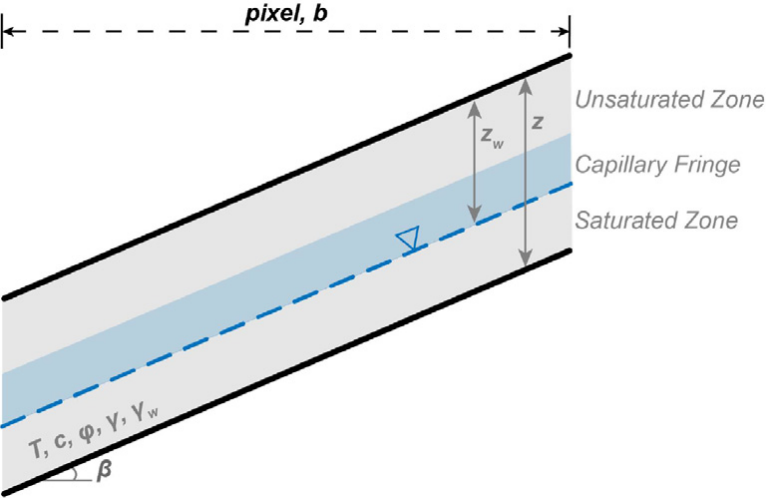
Geometry of the shallow landslide initiation model. Modified after Baum et al. [65].

MM_3_ uses the Fortran based software TRIGRS to compute transient changes in the water table in response to precipitation represented as intensity-duration pairs [65]. TRIGRS uses the one-dimensional Richards equation to describe vertical rainfall infiltration through a two-layer, unsaturated-saturated soil profile divided by a capillary fringe (Fig. 10). A simple runoff routing procedure diverts excess water from less permeable grid cells to more permeable cells downslope. One of the optional outputs of TRIGRS is a three-dimensional grid of pore pressure at user-specified depth intervals [[47], [65]]. MM_3_ utilizes this output to calculate changes in water table depth as the driver of precipitation-induced landslides.

##### Debris flows/avalanches

The static factor of safety of against shallow, planar slides is calculated with the classic infinite slope equation, modified to include the effects of root cohesion [66]:

(9) $$FS=\frac{\left(c+c_{r}\right)+\left(\gamma z\cos^{2}\beta -u\right)\tan\varphi }{\gamma z\cos\beta \sin\beta }$$

where *FS* is the factor of safety, *c* is cohesion, *c_r_* is root cohesion [kPa], *γ* is unit weight of the soil [kN/m^3^], *β* is the slope angle [rad], *φ* is the friction angle [rad], and *u* is the pore pressure acting on the failure plane, $u=\gamma_{w}\left(z-z_{w}\right)$. *γ_w_* is the unit weight of water [kN/m^3^].

The volume of a single failing cell is the product of its plan area and the failure depth, *z*. Adjacent failing cells are clustered in a GIS environment to identify the spatial extent and volume of individual shallow landslide sources.

##### Rockfalls

**Fig. 11:**
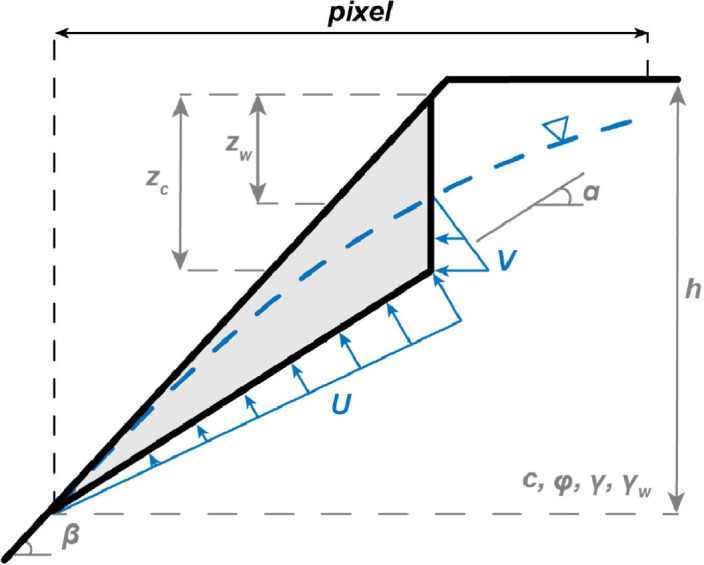
Geometry of precipitation-induced rockfall model.

Rainfall-induced rockfalls are modeled as Culmann wedge-like masses with a tension crack in the slope face [67], including the effects of pore-pressure acting in the tension crack and on the failure plane (Fig. 11):

(10) $$FS=\frac{cA+\left(W\cos\alpha -U-V\sin\alpha \right)\tan\varphi }{W\sin\alpha +V\cos\alpha }$$

where *W* is the weight of the sliding block:

(11) $$W=\;\frac{1}{2}\gamma H^{2}\left[{\left(1-\frac{z_{c}}{h}\right)}^{2}\cot\alpha *\;\left(\cot\alpha \tan\beta -1\right)\right]$$

*U* is the pore pressure acting on the sliding plane:

(12) $$U=\;\frac{1}{2}\gamma_{w}z_{w}\left(h+b\tan\beta -z_{c}\right)\csc\alpha$$

and *V* is the pore pressure acting in the tension crack

(13) $$V=\;\frac{1}{2}\gamma_{w}{z_{w}}^{2}$$

*FS* is the factor of safety of the rock wedge, *γ* is the unit weight of rock [kN/m^3^], *h* is the height of rock face [m], *β* is the slope angle [rad], *φ* is the friction angle [rad], *γ_w_* is the unit weight of water [kN/m^3^], *z_w_* is the depth of the water table [m], *c* is the cohesion [kPa], *z_c_* is the critical tension crack depth [m], and *α* is the critical failure plane angle [rad]. The height of the failing wedge is constrained to 35 % of the local relief based on observations from past earthquakes and calibration on a coseismic landslide inventory from New Zealand [[12], [68]]. We assume that the critical tension crack will initially develop under dry conditions, in which case, depth *z_c_* is:

(14) $$z_{c}=\;\frac{1}{h}\left(1-\;\sqrt{\cot\beta \tan\alpha }\right)$$

after Wyllie and Mah [67]. The critical failure plane angle is typically $\alpha =\;(\frac{\beta +\;\varphi }{2})$ [67]. However, this assumes the absence of water and is inexact for low slopes, especially when there is cohesion (e.g., due to mineral bonding; [69,70]). The critical angle occurs when the factor of safety is at a minimum, or δ_FS_/δ_α_ = 0. MM_3_ solves for the minimum graphically for the range of susceptible slope values and creates a regression relationship between *α* and *β* for each unit, described by a unique set of material and topographic parameters and each ground water configuration.

The rock wedge model in Eq. (10) represents idealized failure geometry and combines individual pixel properties with larger hillslope conditions. As such, the volume associated with Eq. (11) encompasses a slope-parallel slice of the larger hillslope at each cell. After adjacent failing cells are clustered, the total rockfall volume is estimated by the product of the average wedge volume and the width of the hillside involved.

##### Rotational slumps

**Fig. 12:**
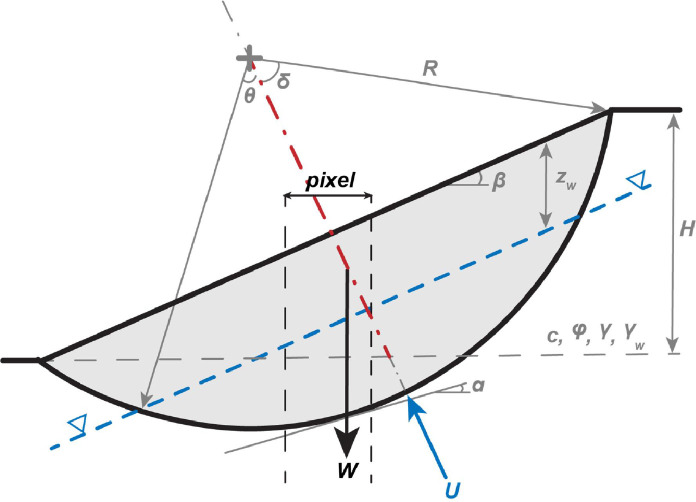
Geometry of rotational slump model. The depth of failure is determined as a percentage, *p*, of the perpendicular hillslope bisector segment in red. *p* may be greater than 100 % (as shown).

Although limit equilibrium slope stability methods for non-planar failure surfaces are well-established in geotechnical practice [66], their implementation in a grid-based GIS framework is challenging due to the computational expense of iteratively testing multiple failure surfaces in each pixel. Both commercial and open-source three-dimensional slope stability programs have been produced (e.g., Slide^3^, [71]; Scoops3D, [47]), but these can only be coarsely implemented at greater than watershed scale (e.g., [72], 2015). Pollock et al. [11] develop an efficient, three-dimensional, pixel-based rotational failure model that computes the limit-equilibrium FS for a uniform, idealized failure surface that combines larger hillslope conditions with individual pixel properties, such as cohesion and friction angle (Fig. 12). The efficiency of the model comes from the limiting assumption of a single prescribed failure surface. The location of the critical failure surface, defined by a depth parameter, *p*, is determined graphically, similar to the regression process to determine *α* for the rockfall model.

To account for wet conditions, the water pore pressure is added to the factor of safety equation, based on a slope-parallel ground water table. MM_3_ projects the radius of failure, *R* [m], perpendicular to the slope to create three-dimensional geometry:

(15) $$FS=\;\frac{2\pi Rbc+\left(W\cos\alpha -F_{w}\right)\tan\varphi }{W\sin\alpha }$$

where *c* and *φ* are the cohesion [kPa] and friction angle [rad] of the soil mass, and α is the basal angle [rad] given by $\alpha =\sin^{-1}\frac{\left(4{\left(\sin\delta \right)}^{3}\sin\theta \right)}{3(2\delta -\sin2\delta )}$. The weight of the sliding block, W, is

(16) $$W=\;\frac{\pi \gamma b^{2}}{3}\left(3R-b\right)$$

and the resultant force of the pore water pressure, $F_{w}$, is:

(17) $$F_{w}=\;\frac{1}{3}\pi \gamma_{w}q^{2}\left(3R-q\right)$$

where *b* and *q* are defined as b=R(1−cos⁡δ) and q=R(1−cos⁡θ). The radius of failure is a function of local relief:

(18) $$R=\frac{H}{4}\left(\frac{\cos\beta }{p{\left(\sin\beta \right)}^{2}}+\frac{p}{\cos\beta }\right)$$

where *H* is local hillslope relief [m] and *p* is the depth parameter. The internal angle, δ, is computed as $\delta =\sin^{-1}\frac{H}{2R\sin\beta }\;$ [rad] for slopes $10^{\circ }\leq \beta \leq 35^{\circ }$, and θ is determined by the water table depth. The volume of overlapping failures is averaged in a similar fashion to that for rockfall failures.

Each model is applied on a pixel-by-pixel basis, producing raster maps of the factor of safety against each mode of failure. Factors of safety below one are assumed to be failing. Due to the overlapping susceptibility zones, individual cells may be modeled as simultaneously failing in two modes under the same input conditions. This coarsely represents the realistic cases of collocated landsliding, such as shallow sloughing in the over-steepened headscarp of a reactivated rotational slump.

#### Coseismic landslide models

For coseismic landslides, we utilize the same equations as for precipitation-induced landslides (Eqs. (9), (10), and (15)). Dry season conditions are modelled either with a deeper groundwater table when seasonal groundwater data is available for use in Eq. (6) or by omitting the pore pressure terms altogether.

##### Coseismic displacements

Static factors of safety for all modes are converted to critical accelerations using the adapted Newmark [73] equation of Chien and Tsai [74]:

(19) $$k_{y}=\frac{FoS-1}{\frac{1}{\tan\varphi }+\tan\alpha }$$

where α is the inclination of the failure plane (equal to *β* for shallow failures) and *φ* is the material friction angle. Eq. (17) is valid for planar and wedge type failures as well as for deep, rotational failures in which the failure plane intersects the slope crest and toe, as in Fig. 12 [74].

Coseismic displacements, *D_N_* [cm], are estimated from PGA after Saygili and Rathje [75]:

(20) $$\ln D_{N}=5.52-4.43\left(\frac{k_{y}}{PGA}\right)-20.93{\left(\frac{k_{y}}{PGA}\right)}^{2}+42.61{\left(\frac{k_{y}}{PGA}\right)}^{3}-28.74{\left(\frac{k_{y}}{PGA}\right)}^{4}+0.72\ln PGA$$

Other displacement relationships can be used (e.g., [76]), including multivariate equations if additional data is available [[75], [77]]. For rotational slumps, coseismic displacements are estimated using Eq. (20), with a 70 % reduction factor applied to the PGA to account for the compliant (non-rigid) nature of deep failure masses [10].

##### Topographic amplification

PGA strongly influences the location of coseismic landslides [78]. Compilations of coseismic landslide inventories show that landslides tend to cluster at ridge crests and near breaks in slope within a broader valley. This spatial “preference” of coseismic landslides is attributed to topographic amplification earthquake ground motion, which produces more intense shaking at topographic irregularities [[68], [78], [79], [80]].

Observationally, a suite of ground motions from five earthquakes in Japan showed that ridgelines experienced a 2.5-fold increase in the intensity of shaking when compared to the shaking at their base [81,82]. Similar studies in Italy and New Zealand recorded base-to-crest amplification factors typically between 2 and 7 [83]. We adopt a fixed topographic amplification term of 1.6 for rock slope failures based on the literature and calibration of MM_3_ using a well-documented coseismic landslide inventory in New Zealand [[12], [84]].

##### Coseismic displacement thresholds

The threshold level of displacement that will produce catastrophic failure varies by slope material and thickness of the shear zone and is not easily determined [85]. In MM_3_, catastrophic failure is assumed at coseismic displacements of 5 cm, 5 cm, and 15 cm for shallow slides, rockfall, and rotational slumps, respectively, after Grant et al. [10].

#### Runout modeling

##### Runout routing using r.randomwalk

**Fig. 13:**
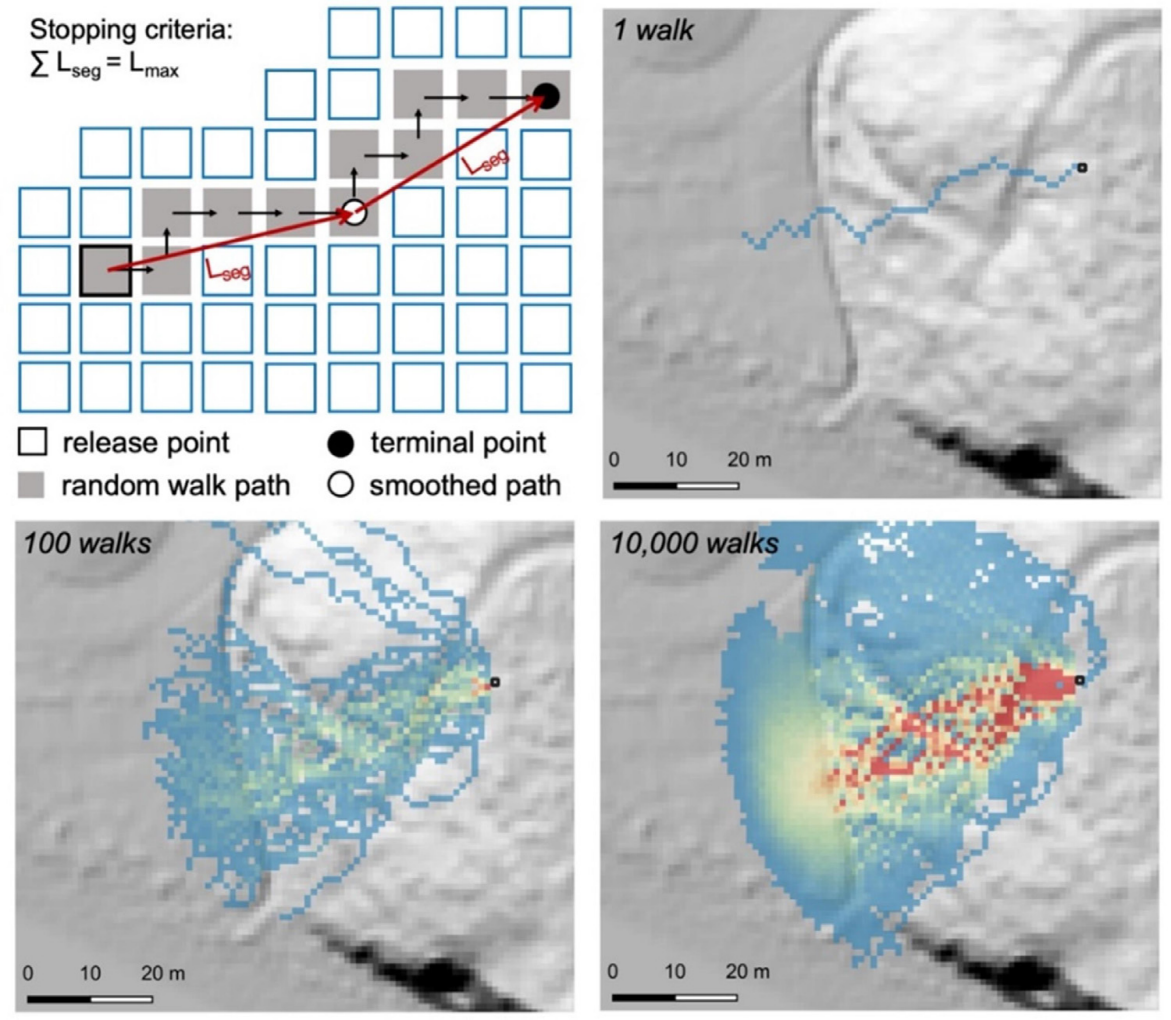
Runout routing in r.randomwalk. The total runout length L_max_ is calculated along a smoothed path by summing increments of the user-defined length L_seg_. After Mergili et al. [86].

MM_3_ maps the runout envelope of shallow soil failures and rockfall using the open-source landslide runout code r.randomwalk [86]. A random walk is a Monte Carlo approach to routing an object through space [87,88]. For landslide runout, flow paths start at user-defined source cells and are built pixel-by-pixel using quasi-random selection from among the adjacent cells (Fig. 13). The selection of cells is constrained by weighted terrain factors including the steepest downhill descent (f_β_) and the perpetuation of flow direction (f_d_). Routing continues until a break criterion is reached such as a reach angle (a.k.a. fahrboschung) or a horizontal travel distance, L_max_ (see Section 2.6.2).

The r.randomwalk code aggregates the random walks from each source cell into a map of impact frequency, describing the number of times a downslope cell is crossed by a flow path (Fig. 13). The runout envelope is defined by all cells crossed by a least one random walk. Uncertainty in the weighting factors f_β_ and f_d_ which control the lateral spreading of individual random walks is accounted for by random uniform sampling between the optimized ranges of 7 to 10 and 1 to 3, respectively [86].

##### Runout stopping criteria

Attempts to quantify and predict the runout length of landslides date at least as far back as Albert Heim's seminal 1932 work, *Bergsturz and Menschenleben* (“Landslides and Human Lives”). Heim advanced the theory that a landslide's runout is fundamentally driven by its volume. Since then, numerous empirical runout relationships have been proposed, the majority of which take one of two forms: (1) relating volume (*V*) to fahrboschung, which is equivalent to the ratio between the vertical and horizontal distances from landslide headscarp to toe, *H_max_* and *L_max_*, respectively [89,90]:

(21) $$\log\left(\frac{H_{max}}{L_{max}}\right)=a*\log V+b$$

or (2) relating volume solely to the total length [91]:

(22) $$\log\left(L_{max}\right)=a*\log V+b$$

An extended discussion of these runout relationships is given in Pollock [12]. The coefficients in Eqs. (21) and (22) are produced via ordinary least-squares (OLS) regression on log-transformed pairs of volume and length (or fahrboschung). For runout prediction, length and fahrboschung are required in arithmetic, not logarithmic space. Eqs. (21) and (22) can be anti-logged to take the power law form:

(23) $$L_{max}=V^{a}*\;10^{b}$$

with a parallel form for Eq. (21). However, performing OLS regression on log-transformed data produces a biased equation when the dependent variable is anti-logged [92,93]. Eq. (23) will underestimate L_max_, with the degree of underestimation increasing with greater scatter in the input data [89,94]. Of particular import for landslide runout prediction is that this bias is non-conservative, and often significantly so, due to a high degree of variability in runout lengths across diverse classes of landslides.

Finney [93] proposed a correction factor (FCF) for least-squares regression on log-transformed data. Eqs. (21) and (22) implicitly assume the statistical model:

(24) $$\log\left(L_{max}\right)=a*\log V+b\;\pm \;\varepsilon$$

where ε is independent, additive, normally-distributed error with mean zero and variance of $\sigma^{2}$. Thus, the anti-logged form is:

(25) $$L_{max}=V^{a}*\;10^{b}*\;\eta$$

where the multiplicative errors, η, are lognormally distributed, of the expectation:

(26) $$\eta \;=\;e^{\left\{\frac{\sigma^{2}*\;{\left(\ln10\right)}^{2}}{2}\right\}}$$

which simplifies to:

(27) $$FCF=\;\eta \;=\;e^{\left\{2.65\;*\;\sigma^{2}\right\}}$$

for base ten-transformed data, where σ is the standard error of the estimate (SEE) in log units [92,94]. Thus, η is greater than one unless there is no scatter around the regression line ($\sigma^{2}=0)$.

Thus, the bias-corrected, predictive form of Eq. (23) is:

(28) $$L_{max}=V^{a}*\;10^{b}*FCF$$

##### Global empirical runout relationships

**Fig. 14:**
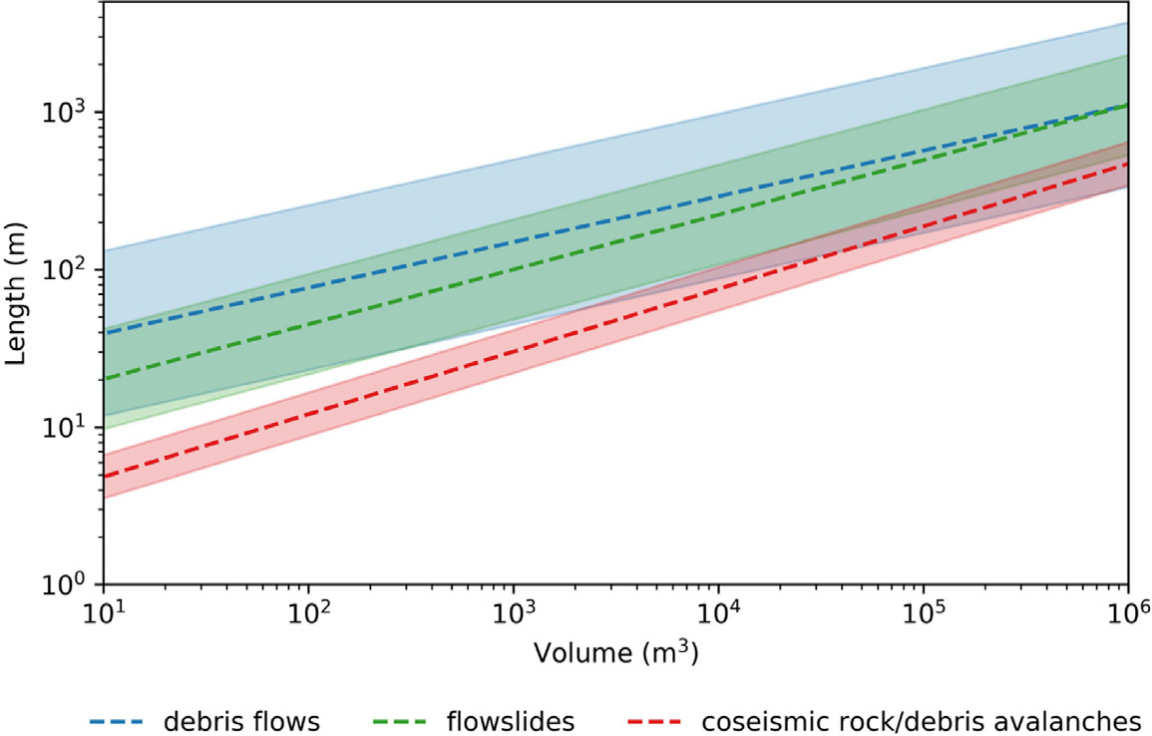
Default length-volume runout relationships in MM_3_. Shaded area shows one standard deviation around the mean.

In many locations, sufficiently detailed inventories of local landslides are not available from which to produce predictive runout relationships that supply the stopping criteria for r.randomwalk. Pollock [12] compiled global datasets of three modes of long-runout landslides to provide preliminary estimates of runout lengths for regions with sparse local data, including coseismic rock avalanches and disrupted soil slides, confined debris flows, and unconfined debris avalanches (sometimes called flowslides). A description of the datasets and the methods used to compile them are given in Pollock [12]. MM_3_ uses the parameter L_max_ as the stopping criteria for r.randomwalk, as it generally has a stronger correlation of landslide volume than does fahrboschung. However, the runout module of MM_3_ allows for other empirical relationships to be selected such as those of Corominas [89] and Rickenmann [95]. Following the form of Eq. (22), the regression coefficients for each mode are given in Table 4. These relationships are visualized in Fig. 14.

**Table 4:** Length-volume relationship parameters.

*Mode*	*a*	*b*	σ	*r^2^*	*n*
coseismic disrupted slides and rock avalanches*	0.3931	0.3269	0.1479	0.83	3999
flowslides (unconfined)	0.3476	0.9578	0.3174	0.92	577
debris flows (confined)	0.2369	1.7472	0.5334	0.30	400

⁎ No statistically significant difference was found between disrupted soil slides and rock avalanches in the dataset of Pollock [12].

##### Channelized and un-channelized terrain

Debris flows typically originate in bedrock hollows or quickly enter a channel after an initial open-slope failure. MM_3_ differentiates between confined debris flows and unconfined debris avalanches by the terrain morphology around the source area. Ten landform types are identified from the base DEM using r.geomorphons [30]. Landslide source areas in confined landforms such as pits, valleys, and hollows are categorized as debris flow-forming while the remaining seven landforms (flats, peaks, ridges, shoulders, spurs, slopes, and footslopes) are associated with debris avalanches.

##### Rotational slumps

Rotational slumps are not considered to have significant runout in MM_3_. The area of influence of each rotational slump is delineated by a spatial buffer equal to the radius of failure, *R* (Eq. (18)). By creating a buffer of *R* rather than the oblique projection of the failing ground surface in Fig. 12, some downslope displacement at the toe and upslope retrogression at the head is assumed. It is possible for rotational slumps to transition into long runout flowslides (e.g., [22]). Such cases could be modeled in a future version of MM_3_ by including a module for evaluating flow liquefaction triggering criteria [12].

#### Process intensity

##### Shallow slides and rotational slumps

The vulnerability of elements at risk is a function of the intensity of the hazard. Various intensity metrics have been proposed for evaluating landslide vulnerability including volume, kinetic energy, velocity, flow depth, intensity index, and momentum flux [[19], [96], [97], [98]]. For rotational slumps MM_3_ uses volume for the process intensity [3], which can be calculated from Eq. (16) by omitting the term for unit weight. The literature typically reports shallow landslide intensity as flow height [99,100]. Since r.randomwalk is a statistical rather than process-based model, it only provides information about the areal extent of landslide inundation. Therefore, MM_3_ makes the generally conservative assumption that the maximum downslope flow height is approximated by the depth of failure in Eq. (9).

##### Rock avalanches

As noted in Section 2.1, large rockfalls and slides can disintegrate into rock avalanches. The post-failure motion of a rock avalanche is rapid and highly destructive, making predictive modeling of the mobility, areal extent, and intensity of rock avalanche runout a key element in risk analysis [101]. Falling blocks move downslope on individual trajectories governed by the gradient, topographic confinement, slope material, and collisions with the substrate and other blocks. Individual blocks may fragment into multiple sub-blocks, with dispersive trajectories, dramatically influencing mobility and degree of spreading of the rock avalanche [102,103]. Fragmentation is a function of initial block size, velocity, rock strength and elasticity, discontinuities, and the properties of the surrounding terrain or colliding rock fragments [104]. The complexity of the process makes fragmentation extremely difficult to model [[102], [104], [105]], and few hazard analyses even attempt to include it [[103], [104], [106]].

Excluding the effects of fragmentation can have significant consequences for hazard and risk analyses. Ignoring fragmentation in rockfall propagation models overestimates the kinetic energy and mobility of rock avalanche runout. Simultaneously, the probability of impact and spreading of the avalanche may be underestimated since fragmentation creates multiple, diverging trajectories [107,108].

Kinetic energy is the primary input to rockfall and rock avalanche vulnerability (e.g., [[50], [109], [110]]), making the velocity and mass of individual rock fragments necessary products of a rockfall runout model. Since rock fragments are typically smaller than the cell size of an analysis and follow discrete trajectories, there is a probability of elements at risk being impacted (and a corresponding probability of non-impact) that is a function of the number and size of the blocks passing slope-perpendicular sections. Few numerical models exist which provide spatially explicit estimates of these parameters [108], and they are too computationally expensive to implement at greater than local scale. Therefore, we approximate mass, velocity, and number of fragments in MM_3_ by coupling simplified energy-based models for fragmentation [102] and velocity [111].

As a rock avalanche progresses downslope, initial potential energy is converted to kinetic energy, which is dissipated by friction and damping in impacts with the surrounding terrain as well as internal friction and fragmentation. Locat et al. [[101], [102]] describe the energy balance of an entire rock avalanche system at any time during post-failure mass movement:

(29) $$\Delta E_{T}=\;\Delta E_{P}+\;\Delta E_{K}+\;\Delta E_{F}+\Delta E_{D}=0$$

where *ΔE_T_* is the change in total energy, *ΔE_P_* is the change in potential energy, *ΔE_K_* is the change in kinetic energy, *ΔE_F_* is the energy lost to basal friction, and *ΔE_D_* is the energy lost in internal fragmentation. From Eq. (29), energy consumed by friction and disintegration reduces the kinetic energy available to perpetuate motion. Based on nine large rock avalanches, Locat et al. [102] find that approximately 20 % of the potential energy of large rock avalanches is spent in internal fragmentation:

(30) $$\Delta E_{D}\;\approx \;0.2*\Delta E_{P}$$

Eqs. (29) and (30) imply that approximately 80 % of the initial potential energy is lost to friction over the duration of the avalanche, or $E_{F}=0.8E_{P}$. This mimics the effect of the initial failing mass falling from 80 % of actual height with the implicit (unrealistic) simplification that the fragmentation energy is lost at the moment of release.

Using the frictional “sled” model proposed by Heim [111], the velocity of the failing material represented by a rigid block (i.e., ignoring internal deformation) can be estimated at any point along the runout path by

(31) $$v=\;\sqrt{2g\Delta z}$$

where g is the acceleration due to gravity and Δz is the height difference between the center of gravity (CoG) “energy line,” defined by $\frac{H_{CoG}}{L_{CoG}}$, and a point along the runout path. Without an a priori assumption of the deposit geometry permitting a calculation the center of gravity, MM_3_ conservatively approximates the energy line with the fahrboschung line, $\frac{H_{CoG}}{L_{CoG}}\;\approx \;\frac{H_{max}}{L_{max}}$ [112]. Thus, accounting for the artificially lowered fall height due to fragmentation, the calculation of Δz for point i becomes:

(32) $$\Delta z=0.8H_{max}\left(1-\;\frac{L_{i}}{L_{max}}\right)-\;H_{i}$$

**Fig. 15:**
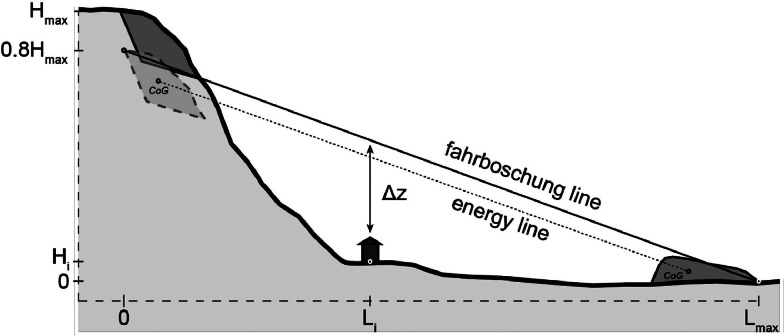
Assuming only 80 % of the original potential energy is available for conversion to kinetic energy and subsequently lost in basal friction, the height difference Δz, and thus velocity, can be computed from an artificial fahrboschung line. Note that this introduces the generally conservative error of using the fahrboschung line instead of the energy line [112].

Where *H_max_* is the total fall height, *L_max_* is the total runout length, and *H_i_* and *L_i_* describe the elevation and distance from the release point to the point of interest (Fig. 15).

By measuring grain size distributions along the transects of nine rock avalanches, Locat et al. [102] empirically derive a relationship between the potential energy per unit volume and the degree of fragmentation (R^2^ = 0.94):

(33) $$\gamma H_{G}=\;\sigma_{c}\left[0.006+0.012\left(\frac{D_{50}}{d_{50}}\right)\right]$$

where *γ* is the rock unit weight [kN/m^3^], D_50_ is the average initial block diameter, d_50_ is the average debris block diameter, s_c_ is the uniaxial compressive strength derived from a point load test, and $H_{G}=\;H_{max}-\;H_{i}$. Eq. (33) can be rearranged to solve for the diameter reduction ratio:

(34) $$R_{r}=\;\frac{D_{50}}{d_{50}}=\;\frac{1}{0.012}\left[\frac{\gamma H_{G}}{\sigma_{c}}-0.006\right]$$

Eq. (34) is used to calculate the block diameter, d_50_, at any point along the runout path as $d_{50}=\;\frac{D_{50}}{R_{r}}$. Diameter is related to the spherical block volume, *V_b_*:

(35) $$V_{b}=\frac{3}{4}\pi \;{\left(\frac{d_{50}}{2}\right)}^{3}$$

Since slope-scale discontinuity spacing is not accounted for in MM_3_, the original block diameter, D_50_, is assumed to equal to the pixel resolution.

**Fig. 16:**
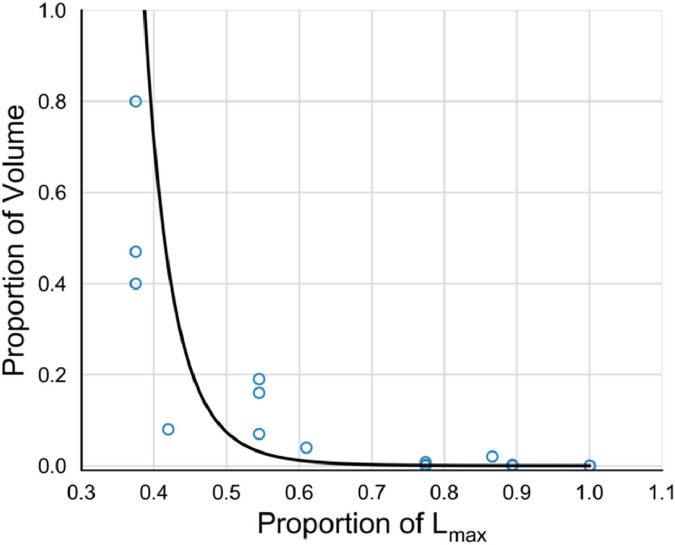
Power law relationship between the proportion of the initial rock avalanche volume passing a proportion of the total runout distance, L_max_ based on four coseismic rock avalanches in New Zealand.

For both rockfalls and debris avalanches, the volume of material passing perpendicular sections downslope can be described by a power law [113]. Based on four coseismic rock avalanches in New Zealand [114], we define a power law relationship between the proportion of the initial volume, *V_L_*, passing a proportion of the total runout distance, *P_L_* (Fig. 16; R^2^ = 0.79):

(36) $$P_{V}=\;10^{-4.19}\;*\;{P_{L}}^{-10.17}$$

This relationship implies that deposition will begin at approximately 38 % of the total runout distance.

From Eq. (36) and assuming an initial cubic block size determined by the pixel resolution, the equivalent number of blocks of mean diameter passing any point along the rock avalanche runout path is:

(37) $$N_{b}=\;\frac{V*P_{V}}{V_{b}}*\;\frac{\pi }{6}$$

The last term, π/6, accounts for a “packing” scale factor that assumes blocks are initially cubic but end spherical due to mechanical rounding. MM_3_ considers only the “design” boulder of mean diameter, d_50_, but future generations of the model could use probabilistic block sizes drawn from real rock avalanche particle-size distributions (e.g., [102]).

Finally, the kinetic energy of each block is calculated as:

(38) $$KE_{b_{i}}=\;\frac{1\;}{2}*\;\frac{\gamma }{g}V_{b}v^{2}$$

Where γ is the rock unit weight, *g* is gravity, $V_{b}$ is the block volume, and *v* is the velocity. Eqs. (29) to (38) represent a simplified approach to approximate the complex dynamics of fragmenting rockfall in a computationally efficient manner. Despite the significant assumptions about the energy loss and geometry of rock avalanche deposits, the kinetic energy model described in this section reasonably imitated a published GIS-based rock avalanche software and an observed rock fall/avalanche in les Cretaux, Switzerland [[12], [115]].

#### Exposure

The exposure of non-stationary elements at risk is typically based on temporal patterns of daily life or traffic volumes (e.g., [[4], [116]]). The temporal exposure of stationary elements at risk is one. However, both stationary and non-stationary elements at risk may have a spatial component to their exposure, depending on the resolution of the study and the magnitude of landslide events (e.g., [117]). For example, in the case of a single falling rock block, the area impacted by the rock and the area occupied by a person may both be only a fraction of the occupied pixel size in a GIS environment. Despite both occupying the same pixel at the same time, the rock and the person may have a low probability of impact, determined as a function of their individual sizes and the pixel resolution [[103], [118]].

Unlike continuum mass-wasting events such as rotational slumps, shallow landslides, or large rock avalanches, small rockfalls are composed of discrete blocks which each pose a unique hazard to elements at risk and may be smaller than the cell resolution of the analysis. It is possible to be within the runout apron of a rockfall without being impacted by debris, as many survivor stories attest (e.g., [119,120]). The probability of impact (*exposure*) is a function of the movement of both the hazard and the element at risk.

For a spatially discrete threat, such as that of being hit by a single falling boulder, the probability of impact on an object within a portion of the slope is:

(39) $$P_{1\left(S:H\right)}=\frac{D+d}{L}$$

where D is the diameter of the design boulder which travels along a path on either side of an element at risk of diameter, d, and L is the length of the slope perpendicular to the runout path [119,121]. The probability of no impact for a single boulder is the complement:

(40) $$1-P_{1\left(S:H\right)}$$

The probability of N boulders passing through L but all missing the element at risk is:

(41) $${\left(1-P_{1\left(S:H\right)}\right)}^{N}$$

so the probability of at least one impact out of N boulders is:

(42) $$P_{N\left(S:H\right)}=1-{\left(1-P_{1\left(S:H\right)}\right)}^{N}$$

Eq. (42) is used to calculate the exposure for each building at risk, with *L* equal to the slope-perpendicular width of the rockfall runout apron defined by r.randomwalk (Section 2.6.1) and d equal to the slope-perpendicular axis of the building [122]. Only indoor populations are considered in MM_3_; the exposure of resident people is interrelated with vulnerability due to the resolution of empirical data, as described below and in Pollock and Wartman [123].

#### Vulnerability

Vulnerability is the degree of loss of an element at risk if impacted by a landslide [5]. In quantitative landslide risk analysis, the inclusion of vulnerability is limited to the physical damage (as a probability of death or a proportion of initial value lost) as correlated to a measurable intensity metric of the landslide hazard such as flow height, velocity, kinetic energy, or momentum [4].

The vulnerability of infrastructure is evaluated economically, expressed through a damage ratio, *D_r_*, between the cost to repair and the cost to replace the structure [[19], [100], [124], [125], [126]]:

(43) $$D_{r}=\;\frac{cost\;to\;repair}{cost\;to\;replace}$$

Vulnerability curves mathematically express the relationship between the damage ratio and the process intensity [99]. Due to the detailed input data requirements and complex nature of landslide-structure interactions, vulnerability curves are usually created using data-driven rather than analytical methods [2]. Numerous curves have been proposed by fitting a chosen mathematical distribution to intensity-damage pairs from observed landslide events (e.g., [[99], [124], [127], [128], [129], [130]]; see review in [19]). Vulnerability is a function of the type and intensity of the landslide as well as building typology, including construction material, foundation type, number of stories, orientation of windows and doors, and maintenance state [131,132], creating the need for unique vulnerability curves for each landslide mode and class of structure [5].

##### Structural vulnerability to shallow landslides and rockfalls

We use the structural vulnerability dataset (*N* = 567) compiled by Pollock [12] to develop vulnerability curves for two landslide modes and two building types: (1) debris flows/avalanches impacting masonry/concrete structures, (2) debris flows/avalanches impacting timber-frame structures, (3) rockfalls impacting masonry/concrete structures, and (4) rockfalls impacting timber-frame structures. An implicit limitation of the underlying data is that almost all observed damage-intensity pairs are for residential structures. Applying vulnerability curves developed with data from smaller, lighter residential structures to dense industrial and urban architecture may overpredict vulnerability and, consequently, risk.

**Fig. 17:**
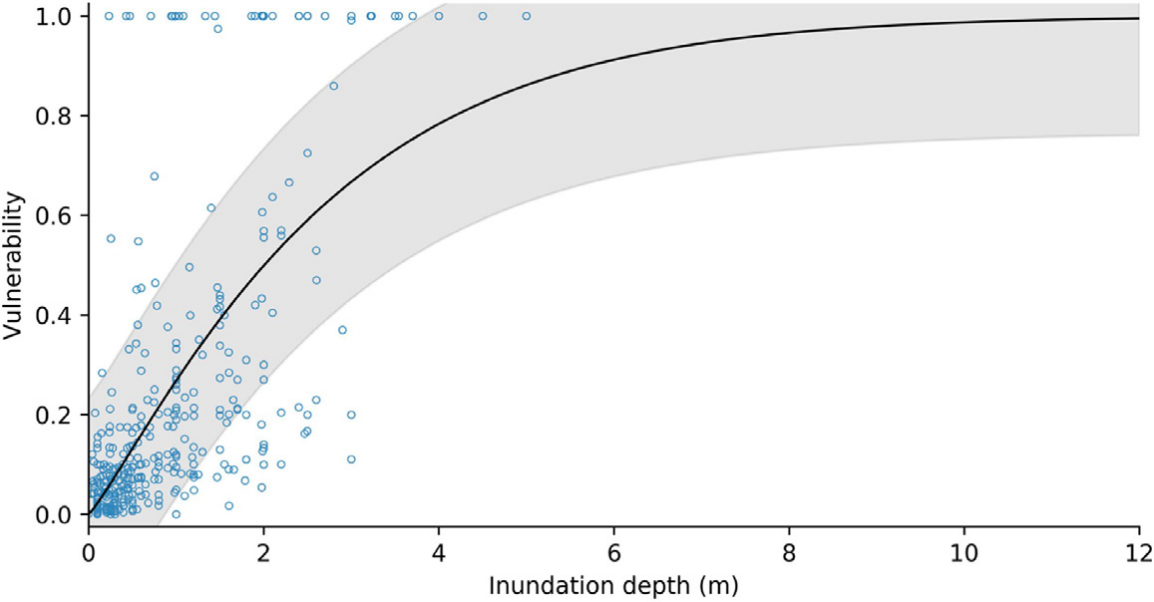
Structural vulnerability curve for masonry and concrete buildings impacted by debris flows/avalanches. Shaded area represents ± one standard deviation.

**Fig. 18:**
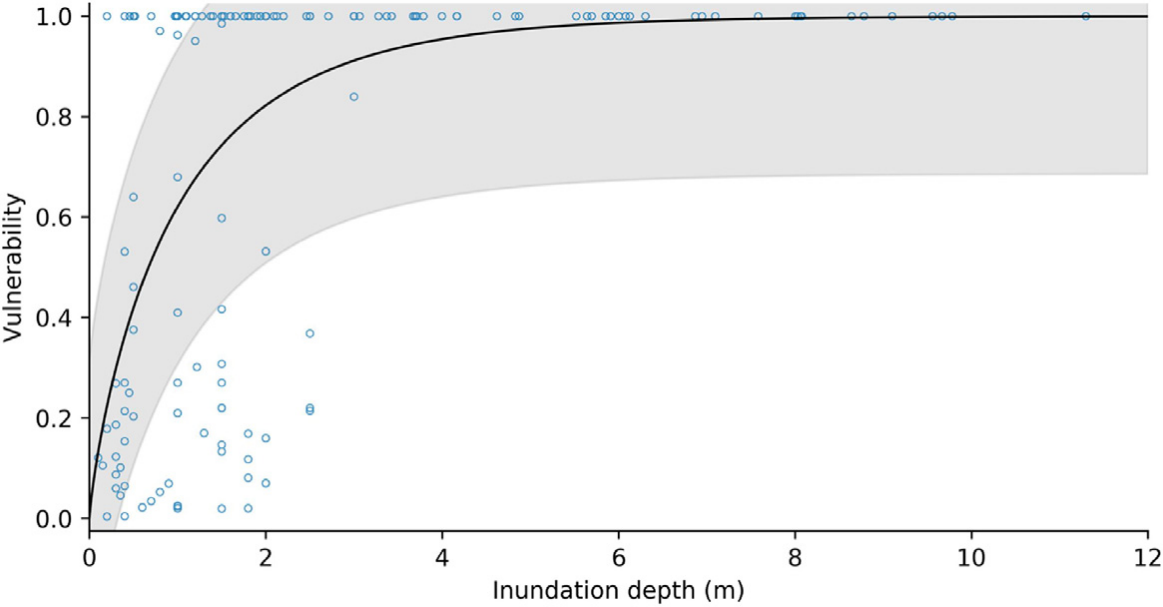
Structural vulnerability curve for timber-frame buildings impacted by debris flows/avalanches. Shaded area represents ± one standard deviation.

Following Totschnig et al. [126] and Papathoma-Kohle et al. [128], we fitted a modified Weibull damage function to the debris flow/avalanche damage-intensity data, of the form:

(44) $$V=1-\;e^{-a\;*\;I^{b}}$$

where I is the debris flow intensity measured in flow depth [m]. Fig. 17, Fig. 18 show the vulnerability curves for masonry/concrete and timber-frame structures, respectively. The fitting parameters for all of the vulnerability curves are provided in Table 5.

**Table 5:** Fitting parameters for structural vulnerability functions. *Standard deviation of the residuals.

*Building material*	*a*	*b*	s*	*pseudo R^2^*	*n*
*Debris flow*					
masonry / concrete	0.3119	1.1470	0.2347	0.40	350
timber	0.9677	0.8359	0.3140	0.33	170
*Rockfall*					
masonry / concrete	1.1073	21.0732	0.2932	0.36	21
timber	0.3239	0.1959	0.2040	0.37	26

**Fig. 19:**
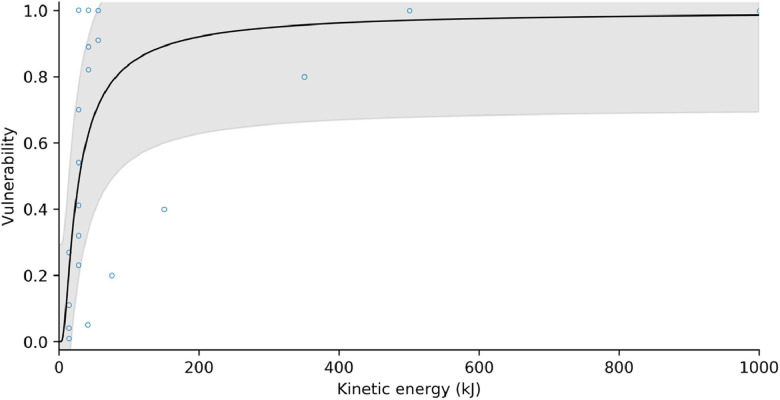
Structural vulnerability curve for masonry and concrete buildings impacted by rockfall. Shaded area represents ± one standard deviation.

**Fig. 20:**
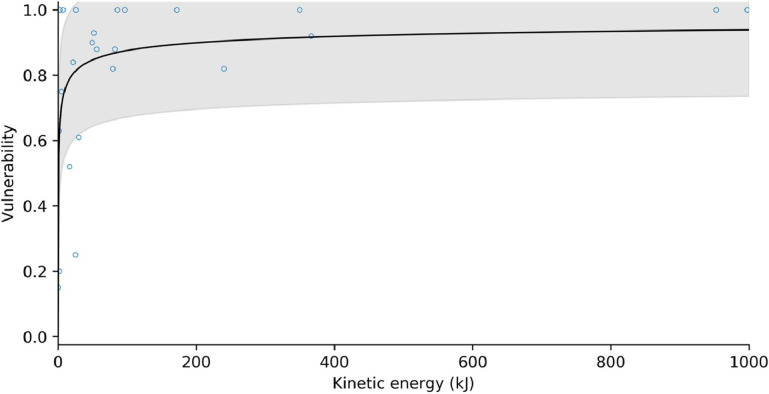
Structural vulnerability curve for timber-frame buildings impacted by rockfall. Shaded area represents ± one standard deviation.

After Massey et al. [19], we fitted a modified Frechet damage function to the rockfall intensity-vulnerability data, of the form:

(45) $$V=e^{-{\left(\frac{I+b}{b}\;-\;1\right)}^{-a}}$$

where I is the rockfall intensity measured in kinetic energy [kJ]. Fig. 19, Fig. 20 show the vulnerability curves for masonry/concrete and timber-frame structures, respectively. Each vulnerability curve is associated with a probability distribution around the best-fit line. In MM_3_, vulnerability values are sampled from this distribution for landslides/rockfall of a given process intensity.

##### Structural vulnerability to rotational slumps

**Fig. 21:**
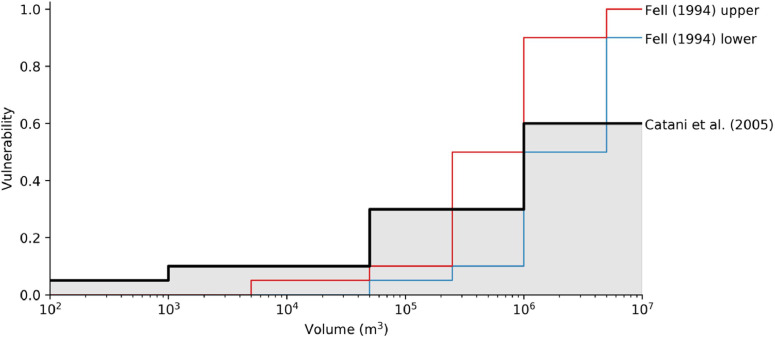
Structural vulnerability to rotational slumps. The values of Catani et al. [134] are used as an upper bound.

Many large rotational slumps are (relatively) slow-moving and experience cycles of increased activity with ground-water fluctuations. Structures wholly within the moving mass may experience little damage if internal deformation is small, while those on the boundaries are subject to differential displacement [133]. Damage to structures on slow moving slumps has been linked to intensity metrics such as landslide area [96], volume [[3], [134]], rate of movement [135], and differential settlement at specific sites on the landslide body [136]. However, the latter two intensity metrics require detailed, long-term monitoring of existing landslides and are difficult to incorporate into predictive modeling of new or dormant slumps. We adopt the volume-based vulnerability values of Catani et al. [134] which is an update of work by Fell [3]; Table 6 and Fig. 21. For slumps of a given volume, vulnerability values are randomly sampled from the shaded area shown in Fig. 21.

**Table 6:** Structural vulnerability to rotational slumps [134].

Volume (m^3^)		Vulnerability
	< 1 × 10^3^	0.05
1 × 10^3^	- 5 × 10^4^	0.10
5 × 10^4^	- 1 × 10^6^	0.30
> 1 × 10^6^		0.60

##### Human vulnerability

Rather than a damage ratio, human vulnerability is often evaluated as the probability of death given direct impact by a landslide or occupancy in an impacted structure [2]. Almost all existing methods to estimate human vulnerability to landslides rely on expert judgement. Pollock and Wartman [123] present an empirical human vulnerability curve for rapid landslides, including debris avalanches and debris flows, which is incorporated into MM_3_. Deep-seated rotational slumps often develop slowly, allowing residents to evacuate [4]; thus we adopt a nominal human vulnerability of 0.0001 in MM_3_. Based on post event interviews after the 2011 Christchurch, New Zealand, earthquake, Taig et al. [120] estimate the vulnerability of individuals in homes struck by rockfalls to be between 0.5 to 0.9 depending on the time of day. Taig et al. [120] observed that because the earthquake occurred in daytime hours, individuals were able to see and evade oncoming boulders, preventing multiple fatalities. Although the ability of humans to take evasive action plays an important role in the estimation of physical vulnerability, it is difficult to predict, as it depends on the specific individual's prior knowledge, awareness, physical ability, and decision-making [123]. MM_3_ assigns values for human vulnerability to rockfalls on a per-building basis by random, uniform sampling within the range 0.5 to 0.9, and no attempt is made to predict *ex ante* the level of human awareness or hazard evasion potential based on time of day.

#### Risk

**Fig. 22:**
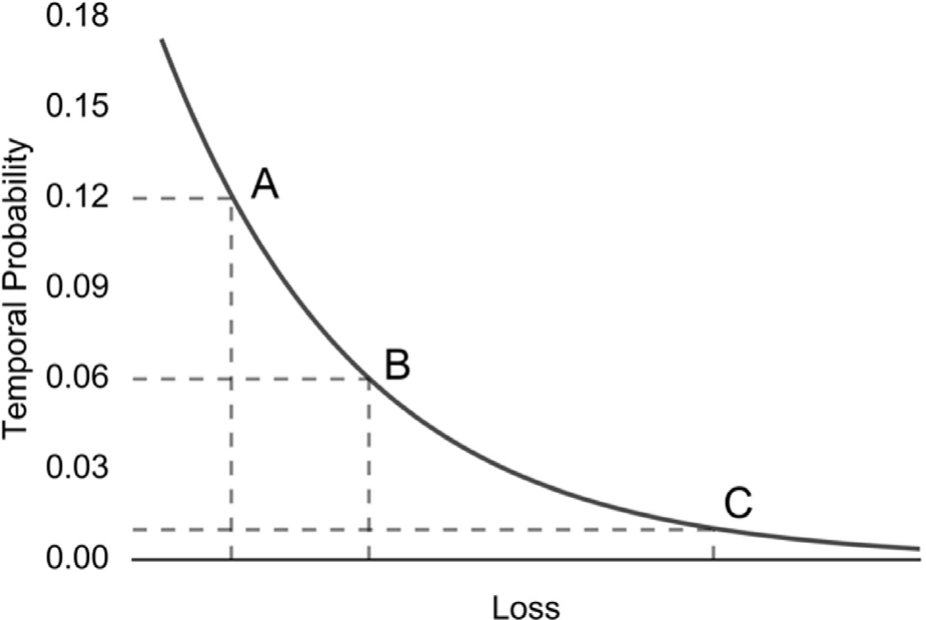
Idealized total risk curve. The three landslide scenarios *A, B*, and *C* are associated with a unique frequency and level of loss. Modified after Corominas et al. [2].

**Fig. 23:**
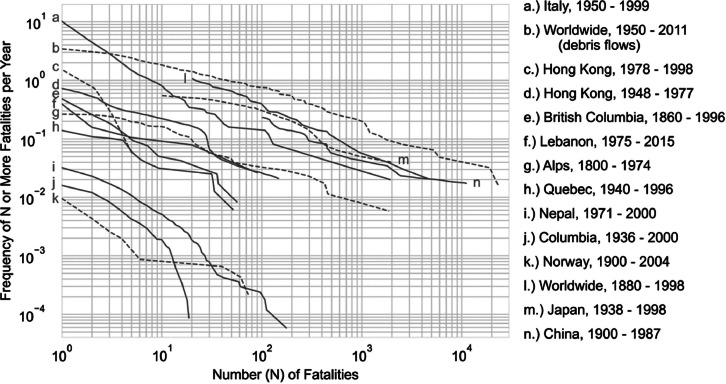
Landslide societal risk “F-N” curves from historical records. Data from Evans [137], Geotechnical Engineering Office [ [138] of Hong Kong (1998], Guzzetti [8], Dowling and Santi [139], and Düzgün and Lacasse [140].

The risk equations (Eqs. (1) and (2)) are applied on a per-pixel or per-object basis, depending on the data format of the elements at risk layer(s), to calculate the loss associated with each triggering scenario and landslide mode. Since each scenario is associated with an annual frequency, the loss values can be summed spatially to plot total risk curves (e.g., Fig. 22) or societal (“F-N”) risk curves (e.g., Fig. 23) or be temporally aggregated into maps of annualized risk. Risk can also be disaggregated to examine the proportion of risk coming from each scenario at a given location. Pollock [12] provides an example of risk disaggregation using MM_3_ in Seattle, Washington, USA.

##### Annualizing risk

The first element of the risk equation (Eqs. (1) and (2)) is the temporal probability of the hazard within a given time period, typically expressed as annual frequency (for events that occur multiple times in a year, such as small rockfalls) or annual probability (for events which reoccur over much longer time periods, such as slumps and shallow landslides). In landslide risk analysis, the temporal probability is derived from a local landslide inventory or equated to the annual probability of a triggering event (e.g., a rainstorm or earthquake) in a physically-based model [2]. Temporal probability can be provided either as a return period or its inverse, annual *exceedance* probability, which should not be confused with the annual *incremental* probability (“frequency”), *h_i_*, in the risk equations (Eqs. (1) and (2); [7]). The exceedance probability of an event of frequency, f, during a given time period, t, is:

(46) $$H_{i}=1-{\left(1-f\right)}^{t}$$

where f is equal to 1/returnperiod [4]. When annualizing risk, the time period of interest is t=1 year and Eq. (46) reduces to the annual exceedance probability:

(47) $$H_{i}=f$$

**Fig. 24:**
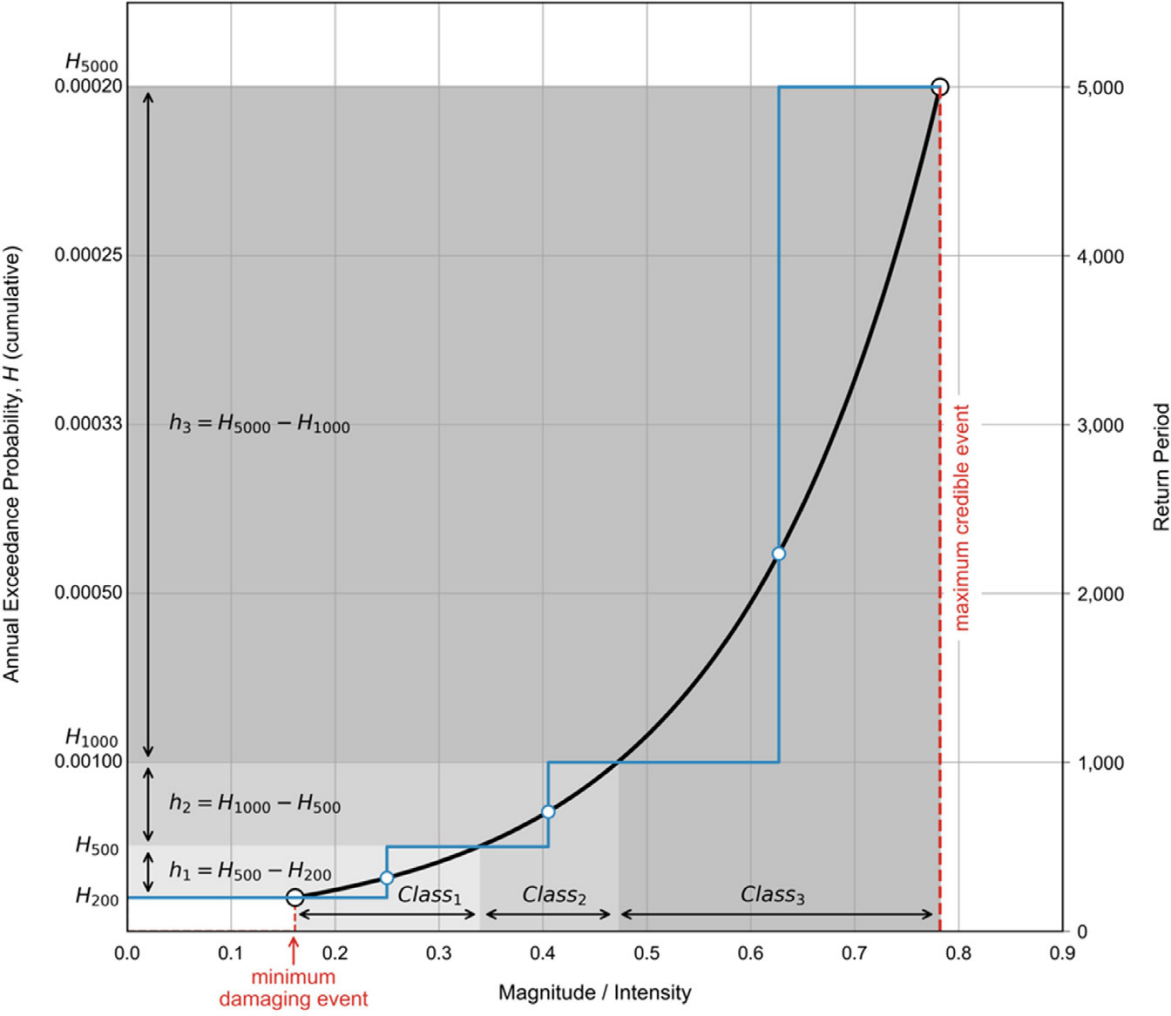
Example of an idealized magnitude-probability curve (black line). In practice the range of plausible event magnitudes is divided into mutually exclusive classes and condensed to a series of representative events (blue circles) used to approximate the idealized curve (blue line). Modified after Strouth and McDougall [7].

The exceedance probability describes the probability of scenario *n* or greater occurring, while the incremental probability describes the probability of exactly scenario *n* occurring [7]. Since there are an infinite number of possible scenarios formed by increasingly fine discretization of landslide magnitude or intensity ranges (or those of the triggering event), it is expedient to assign a single temporal probability to bins of the magnitude/intensity parameter. This can be visualized by a magnitude/intensity-probability (M-P) curve (Fig. 24). An optimized risk analysis uses the fewest number of classes (bins), each represented by a characteristic event, that encompasses the full range of credible damaging scenarios.

Where detailed historical landslide records exist, bins can be heuristically assigned based on observed differences in damage between landslides of different sizes (i.e., such as a fan where channel avulsion occurs only in debris flows over a threshold size). Alternatively, local guidelines may prescribe return periods of interest for which the representative magnitude/intensity range must be determined (e.g., the 100-year and 500-year event). Where records are incomplete or not representative of anticipated future conditions, the latter approach may be the only feasible option.

The incremental probability of scenario *i*, representing all events within class *i* of magnitudes/intensities, is:

(48) $$h_{i}=\;H_{i,\;lower}-\;H_{i,\;upper}$$

where *H_i,upper_* and *H_i,lower_* are the annual exceedance probabilities of the magnitude/intensity class boundaries. Thus, further discretization of the classes into smaller and smaller bins may increase the precision of the risk estimate but does not necessarily increase the total risk since the difference between *H_i=n,upper_* (max credible) and H_i=1_*_,lower_* (minimum damaging) remains constant (Fig. 24).

We note that *h_i_* depends on the subjective selection of magnitude/intensity bin margins, which will vary between risk analysts. Of particular significance is the selection of the minimum damaging event and the maximum credible event. The minimum damaging event is represented by the lowest and leftmost point of the M-P curve (Fig. 24) and is the smallest magnitude/intensity event expected to cause substantive damage. Since the minimum damaging event is associated with high probability (*H_i=1_* is large), it exerts a strong control on total risk. The maximum credible event defines the largest event that could plausibly occur based on site conditions (e.g., slope geometry, geologic control, sediment supply, etc.; [7]). Since the M-P curve tends to approach the maximum credible event asymptotically (i.e., small increases in magnitude/intensity correspond to large decreases in annual exceedance probability), total risk is often insensitive to the exact value of *H_i=n,upper_*. For instance, $h_{1,000-5,000}=\frac{1}{1,000}-\;\frac{1}{5,000}$ is 0.0008, while $h_{5,000-100,000}=\frac{1}{5,000}-\;\frac{1}{100,000}$, is only 0.00019.

#### Probabilistic implementation of MM_3_

Uncertainty can be explicitly modelled in landslide risk analyses through simplified procedures such as the method of moments or through a Monte Carlo process [141]. MM_3_ adopts a Monte Carlo approach. Pollock et al. [142] perform a deterministic landslide risk analysis for the country of Lebanon using an earlier generation of the multimodal method (MM_2_), while Pollock [12] demonstrates MM_3_ in a PLRA for Seattle, Washington.

##### Combining scenarios

As mentioned in Section 2.1, individual cells can “fail” in multiple modes simultaneously. Similarly, elements at risk may be impacted by the same or different modes of landslides across different triggering scenarios and individual Monte Carlo realizations. However, omitting post-disaster reconstruction or reoccupation, each structure can be totally destroyed only once (and each individual can be killed only once). This leads to the questions of which mode, triggering scenario, and model realization is associated with the damage, how damage accumulates, and how to prevent overpredicting risk through cumulative losses greater than the total value (or resident population) of a building. No single “correct” way to combine hazard or risk scenarios exists. Due to the focus on risk of this work, we have adopted a method that aggregates different scenarios *after* the final risk calculation for each building in each Monte Carlo realization. Other approaches could involve aggregating during the calculation of hazard (e.g., [10]).

Within each return period, for each Monte Carlo realization, the three landslide modes are processed separately and then iteratively aggregated into a running mean and variance of risk on a per building basis, according to the method of Welford [143]. For scenario *k* of scenarios *1 – n*:

(49) $$M_{k}=\;M_{k-1}+\frac{\left(x_{k}-\;M_{k-1}\right)}{k}$$

and:

(50) $$S_{k}=\;S_{k-1}+\left(x_{k}-\;M_{k-1}\right)*\;\left(x_{k}-\;M_{k}\right)$$

where *M* is the mean, *x_k_* is the value in scenario *k*, and variance is:

(51) $$\sigma^{2}=\frac{S_{k}}{k-1}$$

for 2≤k≤n.

A running mean and variance are computationally useful as it allows the computer RAM to release each Monte Carlo realization after updating the totals from Eqs. (49) to (51).

**Fig. 25:**
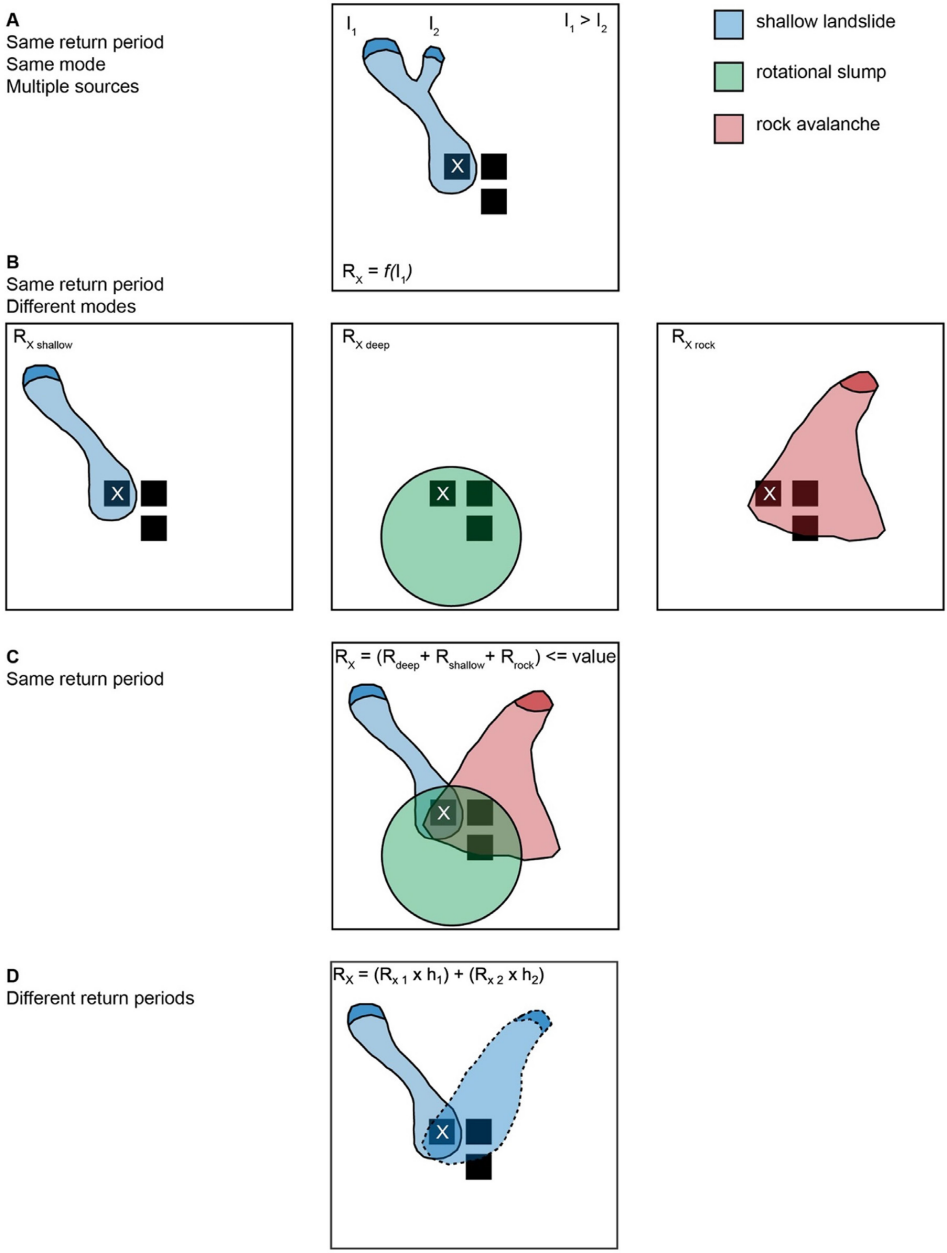
Process for combining risk scenarios. (*A*) in single Monte Carlo run, a runout zone may be associated with more than one source zone. The risk to building X (*R_X_*) is a function of the source zone with the highest intensity. (*B*) In a single Monte Carlo realization, hazard from multiple modes is calculated simultaneously, but not combined, and thus treated as separate scenarios. (*C*) For each building the mean risk from each mode is summed and capped at the total value (or occupancy) of that building. (*D*) The total annual risk for each building is the sum of the annual contributions from each return period, weighted by the associated incremental probability, *h_i_*.

Different modes of failure are combined at the return period (i.e., triggering scenario) level. The risk (human and monetary) is summed across modes at each building and then capped at the maximum occupancy or value of that building. This process is illustrated in Fig. 25. In *panel A*, representing a single Monte Carlo realization associated with one landslide mode, building *X* is struck by shallow landslide runout. The model r.randomwalk produces only the total runout envelope, which may be associated with more than one source zone, each with a unique intensity (i.e., failure depth), *I_1_* and *I_2_*. For that scenario, the risk to building *X, R_X_*, is a function of the maximum intensity, *I_1_.* Panel *B* represents one realization of the Monte Carlo simulation, now showing risk to building *X* from three landslide modes, which is calculated separately. The different modes cannot properly be combined at this level, because the strength parameters used in the factor of safety calculation for each mode are chosen separately, even though they share an initial groundwater condition. Within a single return period of *n* Monte Carlo realizations, Eqs. (46) to [48] calculate the mean and standard deviation or risk separately for each mode. A single realization contributes *1/n* of the total for each mode. For a given return period, the mean risk values (± one standard deviation, etc.) are summed for each building and capped at the total value (or occupancy) of the building (Fig. 25**,** *panel C*). Note that this is more realistic than selecting only the maximum risk of the three modes because it reflects the possibility that in a single triggering event, a building could be impacted by multiple modes of landsliding, the individual consequences of which accumulate to complete loss. Finally, in *panel D*, the annual contributions of each return period event are summed.

This approach implies that, in any given realization, the element at risk could be damaged by all landslide modes, so long as total risk to that building aggregated across all Monte Carlo realizations and modes does not exceed the total value or occupancy of that building. The reasons for aggregating modes at the return period rather than scenario level are both practical and theoretical. Aggregating at the scenario level would be both memory intensive and prevent an accurate disaggregation of the risk contributions by mode. Additionally, sampling of the soil and rock material parameters occurs independently in each scenario. For instance, within a surficial geologic unit, rock strength may be sampled two standard deviations below its mean while the colluvial soil weathered from the same rock unit may be sampled two standard deviations above its mean strength, a physically unlikely case.

## Method validation

A pilot application of MM_3_ in Seattle, Washington, USA, is described in a companion paper. Although comprehensive loss data is not available for specific landslide triggering events in Seattle, Pollock [12] compares the output of MM_3_ to historical landslides and landslide fatalities in Seattle, as well as to city-wide landslide susceptibility mapping in the literature. Pollock [12] tested the sensitivity of components of MM_3_ to variations in user-selected inputs and evaluated the accuracy of MM_3_ in predicting landslides from historical earthquake and rainfall events in Kaikoura, New Zealand, and Portland, Oregon, USA, respectively.

## Limitations

Landslide risk analysis involves significant uncertainty, which can be categorized into two main types: *aleatory* and *epistemic. Aleatory* uncertainty stems from the stochasticity inherent in natural processes, including variations in soil parameters, landslide runout, and the vulnerability of people and buildings. MM_3_ addresses significant sources of aleatory uncertainty by assigning probability distributions to key parameters and modeling risk scenarios using a Monte Carlo approach. However, substantial *epistemic* uncertainty arises from the necessary simplification of complex, real-world phenomena into models. This occurs in every element in Eqs. (1) and (2). We highlight a few examples:

• The temporal frequency of landslide hazard scenarios is dependent on seismic and rainfall hazard analyses, which are subject to their own assumptions, uncertainties, and limitations. Incomplete historic records and long-term changes in climate or seismicity lead to unquantified uncertainty in the timing of follow-on landslide hazard.
• Observationally, only a small and outwardly stochastic subset of slope units sharing similar modeled factors of safety will fail during a given triggering event, demonstrating the strong control of micro-variations in geology, stratigraphy, hydrology, topography, and vegetation. Some of these hyper-local predisposing factors are transient, such as blocked drains, dead root systems, intense storm cells, and recent soil stress history, and almost all are only identified in site-specific, *ex post facto* investigations. One the greatest uncertainties in MM_3_ is the use of surficial geologic maps as a proxy for subsurface conditions. This generalization may be suitably accurate for areas with a relatively simple geologic history but cannot capture buried stratigraphic or structural controls on landsliding.
• Small variations in topography or the built environment (e.g., low retaining walls) can significantly influence landslide runout behavior yet may not be represented in even high-resolution terrain models. Some buildings may be destroyed by landslides while others deflect and channelize debris, altering the runout path. However, determining in which group a specific building belongs is rarely clear prior to the event.
• The built environment changes rapidly. Generally, new construction expands the spatial extent of elements at risk, while retrofitting and urban densification change the use and vulnerability of structures. Population movement may be seasonal, uneven, and sudden, shifting people into or out of landslide hazard areas. For instance, the COVID-19 pandemic increased the time individuals spend at home, both through temporary stay-at-home orders and the proliferation of flexible work-from-home policies. Specific lifestyle patterns of an individual also affect landslide risk as they move through areas of greater or lesser hazard, including time spent in different parts of the home, outside, at work, or traveling.
• The vulnerability of buildings to landslides, and thus also resident people, depends not only the landslide flow (depth, velocity, grain size content, viscosity, etc.) but also on the building orientation, age, state of repair, window layout and height. However, these relationships are, as of yet, largely unquantified.
• Even estimating a “simple” quantity such as structure value is complex. For example, is the “true value” of a structure the current market value, the assessed tax value, the cost of physical building materials (with or without the internal contents such as furniture and appliances), or the full cost of labor and materials to restore a damaged building to pre-event condition? Value estimates may vary significantly between the methods used, individual assessors, and temporal fluctuations in the local economy.

Since the output of each module in MM_3_ is an input for the next, over- or under-estimation of an initial value may be compounded with subsequent steps. MM_3_ is a regional-scale tool, and its efficiency at such scales is facilitated by using remote-sensing datasets rather than site-specific field reconnaissance and subsurface exploration. Granular risk estimates (e.g., building-level) may convey the perception of greater spatial precision than actually exists. Even though sub-meter resolution terrain and satellite data is increasingly available, geologic maps are (and likely will remain) subject to spatial uncertainty on the order of dozens of meters (see Section 2.3.5).

We note that MM_3_ is designed as a screening-level tool intended to guide and complement, rather than replace, site-specific studies and local engineering judgement. Despite inherent uncertainties, MM_3_ offers a first-of-its-kind tool for analyzing landslide risk. It allows for comparison and assessment in relation to other natural hazards, across different locations, and considering various landslide types and triggering processes. MM_3_’s physical basis enables its spatial transferability and applicability to both forward and backward analyses. These analyses encompass probabilistic landslide risk assessment, event back-analysis (e.g., [12]), and scenario modeling (e.g., [50]).

## Ethics statements

The authors have read and follow the ethical requirements for publication in *MethodsX* and confirm that the current work does not involve human subjects, animal experiments, or any data collected from social media platforms.

## Declaration of competing interest

The authors declare that they have no known competing financial interests or personal relationships that could have appeared to influence the work reported in this paper.

## Data Availability

No data was used for the research described in the article.
